# Linker histone regulates the myeloid versus lymphoid bifurcation of multipotent hematopoietic stem and progenitors

**DOI:** 10.1073/pnas.2509412122

**Published:** 2025-10-21

**Authors:** Kutay Karatepe, Bruna Mafra de Faria, Jian Zhang, Xinyue Chen, Hugo Pinto, Dmitry Fyodorov, Esen Sefik, Michael A. Willcockson, Richard A. Flavell, Arthur I. Skoultchi, Shangqin Guo

**Affiliations:** ^a^Department of Cell Biology, Yale University, New Haven, CT 06520; ^b^Yale Stem Cell Center, Yale University, New Haven, CT 06520; ^c^Department of Cell Biology, Albert Einstein College of Medicine, Bronx, NY 10461; ^d^Department of Immunobiology, Yale University, New Haven, CT 06520

**Keywords:** linker histone, myeloid bias, inflammation

## Abstract

Myeloid-biased differentiation of multipotent hematopoietic stem and progenitor cells (HSPCs) occurs with aging or exhaustion. The molecular mechanism(s) responsible for this fate bias remain unclear. Here, we report that linker histone regulates HSPC fate choice at the lymphoid versus myeloid bifurcation. Linker histones package nucleosomes and compact chromatin. HSPCs expressing a doxycycline (dox)-inducible H1.0 transgene favor the lymphoid fate, display strengthened nucleosome organization, and reduced chromatin accessibility at subsets of genomic regions. The genomic regions showing reduced chromatin accessibility host many known marker genes of myeloid-biased HSCs. The transcription factor *Hlf* is located in one of the most differentially closed regions, whose chromatin accessibility and gene expression are reduced in H1.0^high^ HSPCs. Failure to reduce *Hlf* expression in multipotential HSPCs abrogates the H1.0-endowed lymphoid potential. Furthermore, HSPCs display aspartyl protease–dependent H1.0 decreases, especially in response to interferon alpha (IFNα). Aspartyl protease inhibitors preserve endogenous H1.0 levels and promote the lymphoid fate of wild type HSPCs. Thus, our work elucidates a molecular scenario of how myeloid bias arises and uncovers a point of intervention for correcting myeloid skewed hematopoiesis.

## Significance

Myeloid-biased hematopoiesis originated from the myeloid-biased hematopoietic stem cells (My-HSCs) underlie diverse pathologic states ranging from hyperinflammation, cardiovascular disease to cancer, and their prevalence increases with aging. My-HSCs have reproducible and concerted changes in gene expression. However, how my-HSC marker genes become upregulated and which upregulated genes drive this lineage bias remain unknown. This knowledge gap poses significant challenges for correcting the myeloid skewed hematopoiesis, which promises hematopoietic/immune rejuvenation. Here, we report a molecular mechanism governing the myeloid versus lymphoid fate bifurcation by linker histones in the multipotential hematopoietic stem and progenitors, offering pharmacologic opportunities for mitigating the myeloid-biased hematopoiesis.

Myeloid-skewed hematopoiesis underlies inflammation, cancer, and other age-related diseases (1, 2). Myeloid-biased HSCs (my-HSCs) expand during aging; depleting my-HSCs by targeting my-HSC markers rejuvenates the hematopoietic and immune system (3, 4). Myeloid skewing can result from altered signaling from the bone marrow niche. For example, aged niches overproduce inflammatory cytokines such as interleukins and interferons (IFN), which by binding to their cognate receptors on HSPCs activate downstream effectors to promote myeloid differentiation (5–8). Besides cell-extrinsic mechanisms, my-HSCs also display characteristic chromatin/epigenetic changes, such that aged HSCs display more open chromatin regions detectible by ATAC-seq (9, 10) and widened H3K4me3 peaks detectible by ChIP-seq (11). These results suggest that the myeloid versus lymphoid fate choice of multipotent HSPCs could have a molecular basis in chromatin accessibility and that aberrant chromatin opening may favor the myeloid fate. However, how chromatin opening leads to myeloid bias in multipotent HSPCs remains unclear. We address this question by generating a model with an inducible linker histone protein.

Eukaryotic nuclear DNA is packaged into a nucleoprotein complex as repeating nucleosome units, consisting of about 147 bp of DNA wrapped around an octamer of the core histones (H2A, H2B, H3, and H4). A fifth histone, the linker histone H1, associates dynamically with nucleosome core particles at the DNA dyad axis. H1 binding to chromatin stabilizes nucleosomes, increases chromatin folding/compaction, and is associated with a transcriptionally repressed state (12–14). The function of linker histones has been perplexing because genetic inactivation of single H1 genes resulted in no overt phenotypes (15–17) due to redundancy among the multiple H1 isoforms (or subtypes). Further reduction of H1 dosage by simultaneous inactivation of multiple H1 genes did result in embryonic lethality (18–20), in association with derepressed expression of imprinted genes (21, 22) and repetitive elements (23) in embryonic stem cells. Low H1 was also found to result in loss of silencing in interferon-inducible genes in cancer cell lines (24, 25). Overall, while linker histones likely regulate cell identity via modulating chromatin accessibility, whether and how this might happen during cell fate choices in multipotent cells remains unclear.

Within the hematopoietic system, several mature cell types (i.e., post fate commitment) have been examined in mice deficient for multiple linker histones. Mice doubly deficient (DKO) in H1.2 and H1.4 have slightly more circulating eosinophils with all other mature blood cell types being normal (26). H1.2/H1.4 DKO germinal center B cells derepress a self-renewal program and transform into lymphoma on a Bcl2 overexpression background (19). Triple H1 gene inactivation in hematopoietic cells was accomplished by crossing H1.2/H1.4 DKO with a conditional H1.3 allele; mature lymphocytes were reduced, accompanied by their decreased proliferation and increased cell death (20). These results suggest that hematopoiesis can withstand near absence of H1.2/H1.4 and/or H1.3, at least at steady state; simultaneously losing three H1 genes is most prominently reflected as reduced lymphocytes while the erythromyeloid lineages remain largely intact (20). These observations raise the question about linker histone functions in the multipotent HSPCs.

We sought to address this question by modulating linker histone levels using a doxycycline (dox) inducible H1.0 transgene, motivated by the fact that mice deficient in this single linker histone gene had somewhat elevated myeloid colony-forming units (CFU), although that report focused on dendritic cells (27). We also generated a second dox inducible transgene, HMGN1, in the same manner to model functional H1 antagonism. HMGN1 (high mobility group nucleosome binding domain 1) similarly binds at nucleosome dyad while counteracting H1’s chromatin compacting function (28–31). Results from these mouse models demonstrate that H1.0^high^ HSPCs favor the lymphoid fate, accompanied with strengthened nucleosome organization and repression of key myeloid driver genes. We show that the endogenous H1.0 protein level decreases in response to inflammatory signals and is amenable to pharmacologic restoration. We propose a molecular model that could connect inflammation to impaired lymphoid potential of HSPCs via the regulation of H1.0.

## Methods

### Mice and Human Cells.

Mouse work was approved by the Institutional Animal Care and Use Committee (IACUC). HPRT::iH1.0-GFP and HPRT::iHMGN1-mCherry chimeric mice were generated by Yale Genome Editing Center and crossed with B6.SJL-Ptprca Pepcb/BoyJ mice and Rosa26::rtTA (32). Transgene expression was induced by feeding dox drinking water containing 1 g/L doxycycline supplemented with 10 g/L sucrose. All experiments, unless otherwise noted, were performed on 8- to 12-wk-old age and sex-matched mice. Experiments with deidentified human cord blood CD34+ cells were supported by the Yale University Reproductive Sciences Biobank HIC#12696, a component of the Department of Obstetrics, Gynecology & Reproductive Sciences, Yale School of Medicine, New Haven, CT.

### Bone Marrow Transplantation.

Recipient C57BL/6 mice (CD45.2) were purchased from Jackson Laboratories and acclimated for 2 wk. Recipient mice were g-irradiated with one dose of 9.5 Gy. Recipients were injected with 1 × 10^6^ BM cells from each donor. Retroorbital bleeding was performed every 4 wk. BM chimerism was analyzed at the end of each experiment.

### ATAC-Seq.

LSK cells were sorted from X^wt^X^wt^ and X^iH1.0-GFP^X^iH1.0-GFP^ mice treated with Dox water for 3 wk. ATAC-seq library was prepared for 50,000 cells and sequenced on Illumina HiSeq 2500 platform. NRLfinder (33) was used to calculate the nuclear repeat length (NRL). GC content of each chromatin state was determined using mm10 genome and nuc-fi function in Bedtools (34).

## Results

### H1.0 Overexpression in HSPCs Leads to Increased Lymphopoiesis with Expanded MPP4 and CLPs.

**Fig. 1.**
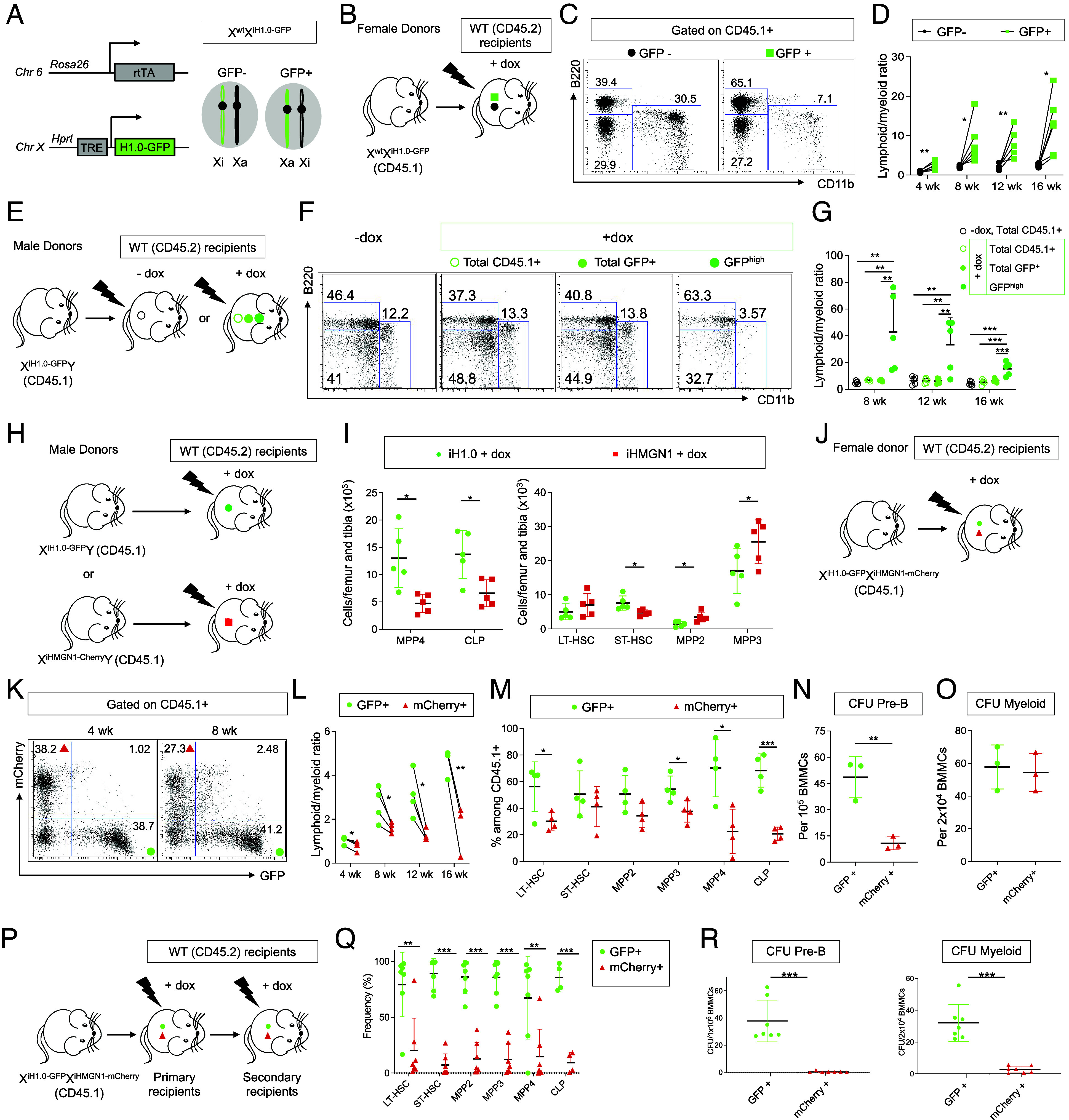
H1.0 expression in HSPCs confers increased lymphopoiesis. (*A*). Diagram for transgene targeting strategy to introduce *H1.0-GFP* into the *Hprt* locus located on chromosome X. *rtTA* is expressed from the *Rosa26* locus. Due to random X chromosome inactivation, heterozygous female cells could have the *iH1.0-GFP* transgene on either X chromosomes. The cells having *iH1.0-GFP* on the active X (Xa), but not those having it on the inactive X (Xi), will show dox-inducible transgene expression. (*B*). Scheme of the experimental setup for noncompetitive whole bone marrow (WBM) transplantation using X^wt^X^iH1.0-GFP^ heterozygous females as donors. All recipients are fed with dox water. (*C*). Representative FACS dot plots for the recipients in *B*. Donor-derived CD45.1 + cells in peripheral blood further divided into GFP- and GFP+ compartments. Representative lineage markers CD11b+ and B220+ cells at 16 wk posttransplantation. (*D*). Quantification of lineage marker-positive cells within donor-derived CD45.1 + cells divided into GFP- and GFP+ compartments as shown in *C*. Lymphoid/myeloid ratio is determined as (%B220+ + %CD3+)/%CD11b+ in individual recipient mice. n = 6 each. (*E*). Scheme of the experimental setup for noncompetitive WBM transplantation using X^iH1.0- GFP^Y male mice as donors. The recipients are treated with regular or dox water. In the dox water-treated recipients, donor (CD45.1 +) cells are further divided by their H1.0-GFP fluorescence intensity. (*F*). Representative FACS dot plots for the recipients shown in *E*, at 16 wk posttransplantation. – dox and + dox total CD45.1 +, total GFP+, and GFP^high^ cells in peripheral blood stained for lineage markers (CD11b and B220) are shown. GFP^high^ denotes the top 10% among total GFP+ cells. (*G*). Quantification of the lymphoid/myeloid ratios in the recipients shown in *F*. (*H*). Scheme of the experimental setup for noncompetitive WBM transplantation using X^iH1.0-GFP^Y or X^iHMGN1-mCherry^Y male mice as donors. The recipients are treated with dox water. (*I*). Quantification of the number of MPP4 and CLP per leg (femur and tibia) in recipient mice 16 wk posttransplantation shown in the *Left* panel; the numbers of LT-HSC, ST-HSC, MPP2, and MPP3 shown in the *Right* panel. n = 5 per group. (*J*). Scheme of the experimental setup for noncompetitive WBM transplantation using X^iH1.0-GFP^X^iHMGN1-mCherry^ females as donors. All recipients are fed with dox water. (*K*). Representative FACS dot plots showing the donor-derived (CD45.1 +) peripheral blood cells at 4 and 8 wk posttransplantation in recipients. (*L*). Quantification of the lymphoid/myeloid ratios within the respective GFP+ and mCherry+ cells within individual recipients. n = 4 for 4 to 12 wk; n = 3 for 16 wk. (*M*). Quantification of bone marrow HSPC subsets positive for H1.0-GFP or HMGN1-mCherry in recipient mice 12 to 16 wk posttransplantation. n = 4 each. (*N*). Quantification of lymphoid colony-forming units by WBM cells at 16 wk posttransplantation. n = 3 each. (*O*). Quantification of myeloid colony-forming units by WBM cells at 16 wk posttransplantation. n = 3 each. (*P*). Scheme of the experimental setup for secondary noncompetitive WBM transplantation using X^iH1.0-GFP^X^iHMGN1- mCherry^ as donors. All recipients are fed with dox water. (*Q*). Quantification of bone marrow HSPCs in secondary recipients 16 wk posttransplantation (n = 4 to 7 each). (*R*). Quantification of lymphoid and myeloid colony-forming units by WBM cells at 16 wk postsecondary transplantation. Individual values as mean ± SD are shown. *P < 0.05, **P < 0.01, ***P < 0.001, by unpaired, 2-tailed Student’s *t* test except for *D* and *L* (paired, 2-tailed Student’s *t* test) and *G* (Ordinary One-Way ANOVA with post hoc Tukey). See also *SI Appendix*, Fig. S1.

We first expressed H1.0 as a GFP-fusion protein by lentivirus in wild-type (WT) Lin- Sca1+ cKit+ (LSK) cells and transplanted them into irradiated syngeneic hosts. HMGN1-GFP and H2B-GFP were expressed as controls, with the former also binding to nucleosome dyad and the latter being functionally neutral and widely used in HSPC tracing studies (35, 36). Following engraftment, GFP+ cells gave rise to distinct lineage contributions: H1.0-GFP+ cells yielded more lymphoid cells (both B220+ B and CD3+ T cells), whereas HMGN1-GFP+ cells had more myeloid progeny (CD11b+) (*SI Appendix*, Fig. S1 *A* and B). The H2B-GFP+ cells had balanced lineages, consistent with the observation that H2B overexpressed as a fusion protein with the fluorescent Timer (FT) in HSPCs did not alter blood lineage distribution (37). To better assess how H1.0 levels regulate HSPC lineage choice, we generated knock-in mice by targeting the coding sequence for H1.0-GFP into the *Hprt* locus under a doxycycline (dox) inducible promoter (37, 38). Upon confirming dox-inducible transgene expression in the targeted mouse embryonic stem cells (mESCs) which express rtTA from the *Rosa26* locus, healthy and fertile mice containing the *X^iH1.0-GFP^* allele (Fig. 1*A*) were derived. Dox-dependent transgene expression in HSPCs was confirmed in LSK cells freshly sorted from iH1.0-GFP mice treated with dox in drinking water for 3 wk (*SI Appendix*, Fig. S1*C*). An *X^iHMGN1-mCherry^* allele was established in parallel as an additional control for transgene targeting as well as for functionally antagonizing H1.

As the *Hprt* locus is located on the X chromosome, dox-treated female heterozygotes (X^wt^X^transgene^) would contain a mixture of transgene+ and transgene- cells, due to random X inactivation. Expressed as fusion proteins, the fluorescence intensity serves as a surrogate for transgene expression levels in live cells (Fig. 1*A*). We first confirmed the increased lymphoid cells from iH1.0-GFP+ HSPCs by transplanting X^wt^X^iH1.0-GFP^ whole bone marrow into lethally irradiated WT recipients, fed them dox water, and followed donor cells over time (Fig. 1*B*). Donor (CD45.1 +) cells fully reconstituted hematopoiesis in WT (CD45.2 +) recipients (*SI Appendix*, Fig. S1*D*), and their contribution to B, T, and myeloid lineages in the peripheral blood (PB) was monitored every 4 wk for up to 16 wk. Consistent with the lentivirally expressed H1.0, transgenic H1.0-GFP+ cells gave rise to increased lymphoid cells, a phenotype strengthening over time (Fig. 1 *C* and *D*). Despite the higher lymphoid reconstitution within the H1.0-GFP+ compartment, all recipients had complete blood cell (CBC) counts within the normal range (*SI Appendix*, Fig. S1*E*), indicating that the H1.0-GFP+ cells follow homeostatic control together with the transgene- cells (Fig. 1*A*). The increased lymphoid contribution by H1.0-GFP+ cells was also seen with male donors (Fig. 1*E*), as shown by increased lymphoid/myeloid ratio in brighter H1.0-GFP+ cells (+dox GFP^high^) (Fig. 1 *E*–*G*). Furthermore and consistent with the lineage distribution observed by the lentivirally expressed transgene (*SI Appendix*, Fig. S1 *A* and B), +dox mCherry^high^ cells derived from iHMGN1-mCherry donor bone marrow displayed decreased PB lymphoid/myeloid ratio (*SI Appendix*, Fig. S1 *F*–H). The opposing lineage effects following HMGN1 overexpression is consistent with the known H1-HMGN1 antagonism (28–31) and the disturbed hematopoiesis in a Down syndrome model of HMGN1 overexpression (39). Together, these results support that the prevalence of peripheral lymphoid lineage cells scales with linker histone levels.

To assess the transgene effects on bone marrow HSPCs, we established cohorts of WT recipient mice engrafted with either X^iH1.0-GFP^Y or X^iHMGN1-mCherry^Y whole bone marrow, fed them with dox water, and analyzed HSPC numbers 16 wk later (Fig. 1*H* and *SI Appendix*, Fig. S1*I*). The lymphoid-biased MPP4, common lymphoid progenitor (CLP), and short term HSC (ST-HSC) were increased in mice engrafted with iH1.0-GFP marrow (Fig. 1*I*), while the myeloid-biased MPP2/MPP3 increased in those engrafted with iHMGN1+ marrow, with minimal changes in the myeloid-committed progenitors (Fig. 1*I* and *SI Appendix*, Fig. S1*J*).

The X-chromosome hosted transgenes afford an opportunity to compare HSPCs expressing either transgene within the same animals, potentially reducing variability and controlling for cell-extrinsic factors. Therefore, we noncompetitively transplanted whole bone marrow from female X^iH1.0-GFP^X^iHMGN1-mCherry^ donors into lethally irradiated WT recipients and fed them dox water (Fig. 1*J* and *SI Appendix*, Fig. S1*K*). While H1.0-GFP+ and HMGN1-mCherry+ cells were similar in abundance at 4 wk (Fig. 1*K* and *SI Appendix*, Fig. S1 *K* and L), %H1.0-GFP+ in all recipients increased over time (Fig. 1*K*), due to increased B and T cells (*SI Appendix*, Fig. S1 *M* and N), yielding higher lymphoid/myeloid ratios in each recipient (Fig. 1*L*). 16 wk after transplantation, the lymphoid-biased MPP4 as well as the lymphoid committed CLP expanded greatly in each recipient, with the iH1.0-GFP+ cells dominating these compartments (Fig. 1*M*); in contrast, changes in MPP2/3 and myeloid-committed progenitors were minimal (Fig. 1*M* and *SI Appendix*, Fig. S1*O*). iH1.0-GFP+ bone marrow cells formed 5x more lymphoid colony-forming units (CFU) than the iHMGN1-mCherry+ cells isolated from the same mice, while myeloid CFUs were similar between two groups (Fig. 1 *N* and *O*). When X^iH1.0-GFP^X^iHMGN1-mCherry^ bone marrows were transplanted into secondary recipients, even the myeloid lineages were primarily reconstituted by H1.0-GFP+ cells, indicating that H1.0^high^ HSCs remain functional at long term (Fig. 1 *P*–*R*). Taken together, elevating H1.0 level leads to increased lymphopoiesis that becomes prominent as expanded MPP4-CLP compartments and results in more B/T cells in the blood. Of note, the endogenous H1.0 mRNA is expressed at high levels in HSPCs and CLPs, as documented by the *Tabula Muris* database (40) due to H1.0 mRNA being polyadenylated (*SI Appendix*, Fig. S1*P*), suggesting that changes in H1.0 level accompany HSPC commitment to the lymphoid fate.

### H1.0-Overexpressing HSPCs Display Reduced Chromatin Accessibility in Subsets of Genomic Regions in Association with Strengthened Nucleosome Organization.

**Fig. 2.**
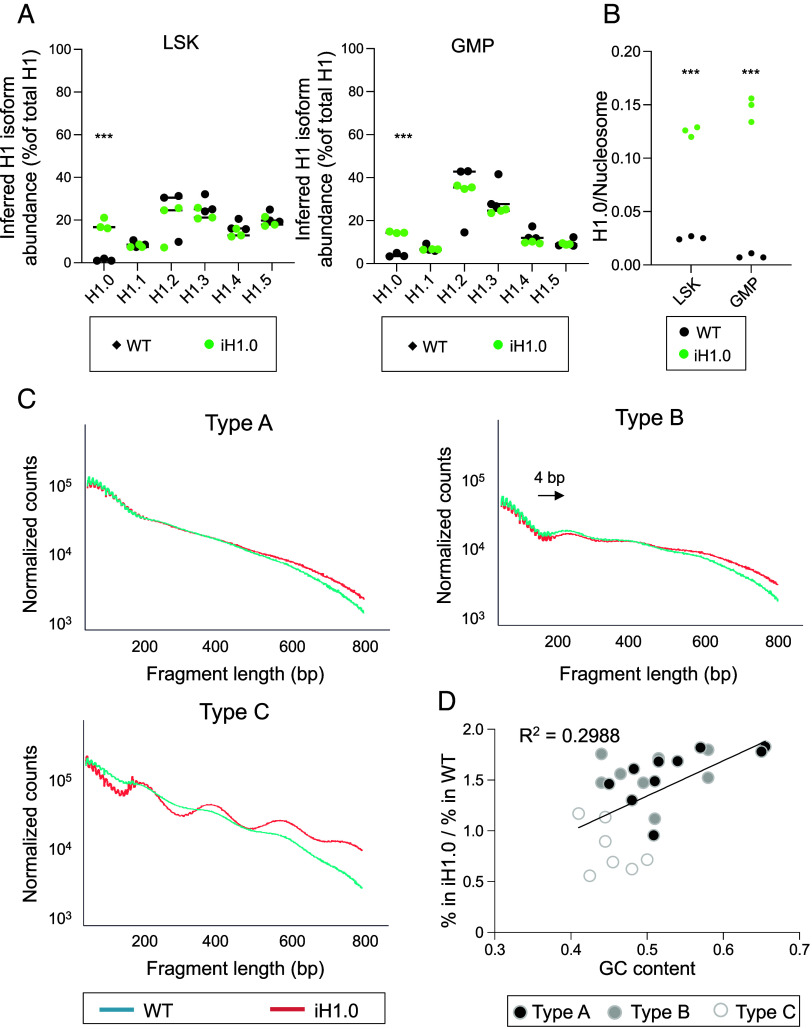
H1.0-overexpressing HSPCs display strengthened nucleosome organization. (*A*). Quantification of the inferred abundance of H1 subtypes as percent of total H1 by LC–MS in WT and iH1.0- GFP+ LSK and GMP cells. n = 3 each. (*B*). Quantification of the inferred H1.0/nucleosome ratio in WT and iH1.0-GFP+ LSK and GMP cells. (*C*). ATAC-seq fragment lengths and density in WT and iH1.0 + LSK cells. Three types of chromatin with regard to their nucleosome repeat signals are detected: Type A contains open chromatin and no nucleosome repeat signal in both WT and iH1.0 LSK cells; Type B has weak nucleosome repeat signal with an average of ~4 bp increase in nucleosome repeat length in iH1.0 + LSK cells; Type C has no nucleosome repeat signal in WT LSK but exhibits strong nucleosome repeats of 190 bp in iH1.0 + LSK cells. (*D*). Linear regression model depicting GC content vs. % of ATAC seq reads in each of the 25 ChromHMM chromatin states in iH1.0 + relative to WT LSK cells. Reads coming from chromatin states with low GC content appear underrepresented in iH1.0 + relative to WT LSK cells. See also Dataset S3 for details. Individual values as mean ± SD are shown. *P < 0.05, **P < 0.01, ***P < 0.001, by unpaired, 2- tailed Student’s *t* test. See also *SI Appendix*, Fig. S2.

Transgenic H1.0 expression is expected to increase H1 protein level to reduce chromatin accessibility (20, 41). We first ascertained increased H1.0 protein in the transgenic HSPCs by liquid chromatography–mass spectrometry (LC–MS) using previously validated triple H1 knockout (TKO) mESCs as controls (20, 22). We found that H1.0 accounted for ~1.3% of the total H1 repertoire in WT LSK cells, a value that increased to ~18% in iH1.0 + LSK cells (Fig. 2*A* and Dataset S1). The abundance of other major H1 isoforms was not significantly altered. Thus, the endogenous H1.0 constitutes a minor portion of the total H1 pool in HSPCs; transgenic H1.0 expression elicited minimal compensation by the other H1 isoforms. Elevated H1.0 resulted in higher H1.0/nucleosome ratio, increasing it from 0.025 to 0.125 in LSK cells (Fig. 2*B* and Dataset S1). Comparable increase in H1.0 protein was detected in GMPs, although no difference was detected in their compartment size (*SI Appendix*, Fig. S1*J*). These results confirm the increase of total H1.0 protein in iH1.0-GFP+ HSPCs; the transgene effect, however, appears to vary among cell types, suggesting that the cellular context may modify the transgene’s effect.

To better understand how increased H1.0-regulated HSPC compartment sizes, we examined nucleosome organization in H1.0^high^ LSK cells. As nucleosomal DNA is less accessible to the Tn5 transposase, the enzyme used in ATAC-seq analysis, plotting the distribution of DNA fragment length in ATAC-seq data could inform nucleosome organization at genomic regions with known nucleotide sequences and chromatin marks (20). We compared ATAC-seq results from iH1.0-GFP+ and WT LSK cells. Each cell type had more than 32,000 ATAC-seq peaks, ~5% of which showed differential accessibility in response to H1.0 transgene with a cutoff of *P* < 0.01 and fold change of >2 (Dataset S2). We focused on the most differentially accessible regions between the genotypes using more stringent cutoffs *P* < 0.001 and fold change of >4. This yielded 242 regions that were more closed in iH1.0 + LSK cells than WT LSK cells, while 78 regions were more open (Dataset S2). The overabundance of closed than open regions in iH1.0 + cells is consistent with linker histone’s chromatin compacting function. The open chromatin regions in the iH1.0 + cells are consistent with the fact that nucleosome density is not uniform across the genome (42). The differentially accessible chromatin regions are similarly distributed across promoters, UTRs, introns, exons, and distal intergenic regions in both WT and iH1.0 + LSK cells, regardless of cutoff stringency (*SI Appendix*, Fig. S2*A*). Of note, ≤ 10% of the closed regions in iH1.0 + LSK cells are annotated as promoters, while the great majority are introns and distal intergenic regions. No enrichment for transcription factor (TF) binding motifs was detected unless the cutoff was relaxed. These results suggest that genome-wide chromatin accessibility remains overall similar in the H1.0^high^ LSK cells, with more pronounced chromatin closing at subset genomic regions.

To further investigate how elevated H1.0 regulates nucleosome organization across the genome, we assessed the nucleosome repeat patterns across a 25-chromatin-state-map defined by ChromHMM using well-validated chromatin/histone marks (43, 44). This analysis yielded three types of chromatin accessibility responses following H1.0 transgene expression (Fig. 2*C* and *SI Appendix*, Fig. S2 *B*–D and Dataset S3). Type A chromatin had minimal nucleosome repeat pattern in either WT or iH1.0 + LSK cells and are similar between the two genotypes (Fig. 2*C* and *SI Appendix*, Fig. S2*B*). Type B chromatin had weak nucleosome repeat signal in WT LSK and the nucleosome repeat signals increased in iH1.0 + LSK cells by ~4 base pairs (Fig. 2*C* and *SI Appendix*, Fig. S2*C*), consistent with more nucleosomal DNA protection by H1. The most significant change is seen in Type C chromatin, in which H1.0 overexpression substantially strengthened the nucleosome repeat patterns (Fig. 2*C* and *SI Appendix*, Fig. S2*D*). Thus, H1.0^high^ LSK cells have reduced accessibility in a small subset of chromatin regions; not all genomic regions respond to H1.0 increase in the same manner.

We next examined the 25 chromatin states to identify features that are associated with the most responsiveness to H1.0 to close chromatin. Surprisingly, a substantial portion of Type C chromatin are designated as quiescent (Dataset S3), i.e., they lack both activating and repressive chromatin marks. In addition, Type C chromatin includes heterochromatins decorated by repressive chromatin marks as well as active chromatins (43). Closer examination revealed that most Type C chromatin appear to contain lower GC (Fig. 2*D*). Considering the previous report that H1.0 prefers high GC regions (45), our results suggest that genomic regions (i.e., Type C) may appear more responsive to increased H1.0 levels due to their inherent low affinity to H1.0; such regions may become more attractive to H1.0 as the linker histone becomes abundant, while regions naturally preferred by H1.0 show little further changes. Taken together, these results could support a nucleosome organization-based explanation for how the lymphoid fate is favored by multipotential HSPCs; critical cell fate regulators may reside in such regions and respond to fluctuating linker histone levels.

### H1.0 Promotes Lymphoid Potential by Reducing Chromatin Accessibility and Gene Expression of *Hlf* in HSPCs.

**Fig. 3.**
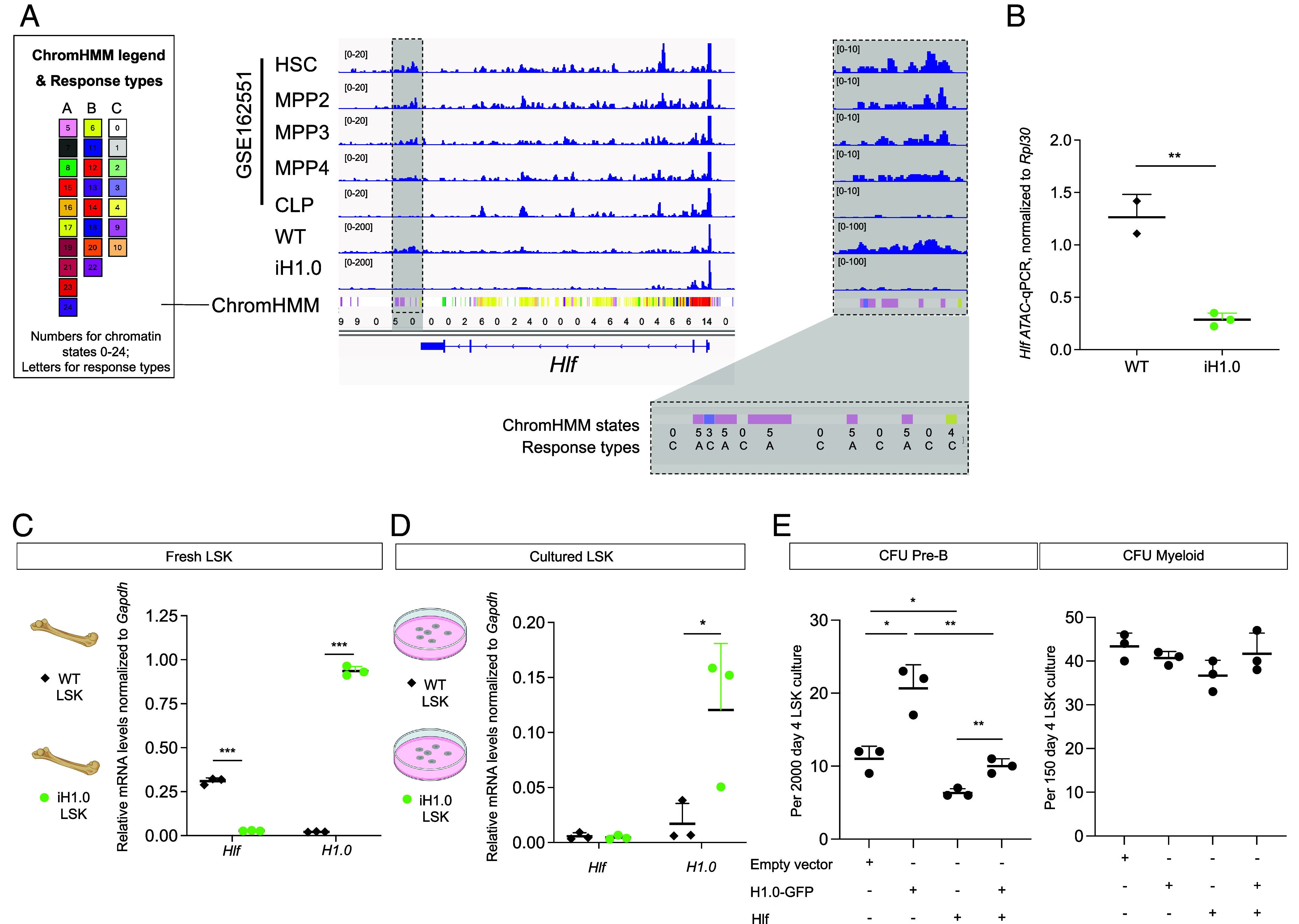
H1.0 expression imparts lymphoid fate potential by reducing chromatin accessibility and gene expression of Hlf. (*A*). Chromatin accessibility measured by ATAC-seq around the *Hlf* genomic region during WT HSPC differentiation from dataset GSE162551, aligned with those obtained from WT and iH1.0 LSK cells, further aligned with the ChromHMM chromatin states. n = 2 for WT and n = 3 for iH1.0 + LSK cells; one representative shown for each genotype. (*B*). ATAC qPCR quantification for the region highlighted in (*A*) from WT and iH1.0 + LSK cells, normalized to that of mRPL30. n = 2 for WT and n = 3 for iH1.0 + LSK cells. (*C*). *Hlf* and *H1.0* mRNA levels in freshly sorted WT and iH1.0 + LSK cells from mice on dox water for 3 wk. n = 3 donors per group. (*D*). *Hlf* and *H1.0* mRNA levels in cultured WT and iH1.0 LSK cells. n = 3 donors per group. (*E*). Quantification of lymphoid and myeloid CFU numbers using WT LSK cells transduced with indicated viral constructs. Each dot is a triplicate sample; results shown are representative of three independent experiments. Individual values as mean ± SD are shown. *P < 0.05, **P < 0.01, ***P < 0.001, by unpaired, 2- tailed Student’s *t* test. See also *SI Appendix*, Fig. S3.

We next inspected the genes located within the closed genomic regions in iH1.0 + LSK cells. As a further control, we aligned our results with a reference ATAC-seq dataset generated during WT HSPC differentiation into lineage-biased or committed progenitors (9, 46). When the 242 most differentially closed chromatin regions in iH1.0 + LSK cells were ranked by the strength of statistical significance and effect size, we found the topmost region to be the *Hprt* promoter, which is the host locus to the transgene (*SI Appendix*, Fig. S3 *A* and B), likely reflecting the chromatin accessibility difference introduced by the transgenic cassette, and no difference is seen during WT HSPC differentiation. The next in ranking was *Tspan9* and *Hlf* (Fig. 3*A* and *SI Appendix*, Fig. S3 *A* and B and Dataset S2). *Tspan9* is mostly expressed by megakaryocytes and platelets (47), while *Hlf* is one of the most well-recognized HSC genes (48–50). In addition, many genes known to be upregulated in my-HSCs (3, 4, 9), such as *Thbs1, Slamf1,* and *Itgb3* had reduced chromatin accessibility in iH1.0 + LSK (*SI Appendix*, Fig. S3*C* and Dataset S2), suggesting that an H1.0-mediated chromatin mechanism could shape the gene expression program that defines the my-HSC identity. Together, these results further support the pro-lymphoid function of H1.0 and nominate key genomic targets underlying the myeloid versus lymphoid fate bifurcation point.

Considering the functional role of Hlf as an HSC reprogramming TF (51), we focused on the potential regulation of *Hlf* by H1.0 to impart lymphoid fate preference. Hlf is a PAR bZip family transcription factor (52) and its expression drives myeloid commitment while interfering with lymphoid differentiation (53). Reduced chromatin accessibility at the *Hlf* genomic region (Fig. 3 *A* and *B* and Dataset S2) could potentially lead to its decreased expression and drive superior lymphoid potential of H1.0^high^ HSPCs. Around the *Hlf* gene, the most prominently reduced ATAC-seq peaks in iH1.0 + LSK cells are an intergenic region ~1 kb 3′ distal to the *Hlf* gene. ChromHMM annotates this region as mostly “quiescent—state 0”, “polycomb—state 3” and “enhancer like—state 4”; interspersed with several “nuclease accessible—state 5” regions (Fig. 3*A* and *SI Appendix*, Fig. S2 *B* and D and Dataset S3). Of note, ChromHMM states 0, 3, and 4 are all Type C chromatin. qPCR with Tn5 fragmented DNA using primers spanning this region confirmed reduced chromatin accessibility in the iH1.0 + LSK cells (Fig. 3*B*). This region and the *Hlf* gene body have ~46% GC (*SI Appendix*, Fig. S3*D*). Strikingly, this region is gradually closed during WT HSC differentiation into the lymphoid-biased MPP4 and almost completely lose ATAC-seq signals in the CLP (Fig. 3*A*). The change in this region’s accessibility during WT HSPC differentiation suggests it to be functional. Indeed, this region contains one ChromHMM state 4 “enhancer-like” region with Type C response, displaying strengthened nucleosome repeats in the H1.0^high^ LSK cells (Fig. 2*C* and *SI Appendix*, Fig. S2*D*).

We next assessed whether *Hlf* expression is altered by the H1.0 transgene by RT–qPCR of iH1.0 + and WT LSK cells. In fresh LSK cells, we found that *Hlf* mRNA was greatly reduced in iH1.0 + cells compared to WT cells (Fig. 3*C*). After 4 d of culture, *Hlf* expression became undetectable regardless of the genotype (Fig. 3*D*), consistent with the loss of *Hlf* expression as HSPCs further differentiated.

To determine whether H1.0 enabled lymphoid potential is dependent on reducing *Hlf*, we expressed Hlf from a viral construct. WT LSK cells were transduced with viral constructs that overexpress either H1.0, Hlf, or both, followed by plating into methylcellulose media permissive for either lymphoid or myeloid colonies (Fig. 3*E*). H1.0 overexpression increased the lymphoid CFU, as expected. However, this increased lymphoid potential was abolished by Hlf coexpression. Similar myeloid CFU were formed in all conditions (Fig. 3*E*). Taken together, these data support the model that H1.0 promotes the lymphoid fate of multipotent HSPCs at least partly by reducing the chromatin accessibility and gene expression of the myeloid driver gene *Hlf*.

### H1.0 Level Is Amenable to Physiologic and Pharmacologic Regulations.

**Fig. 4.**
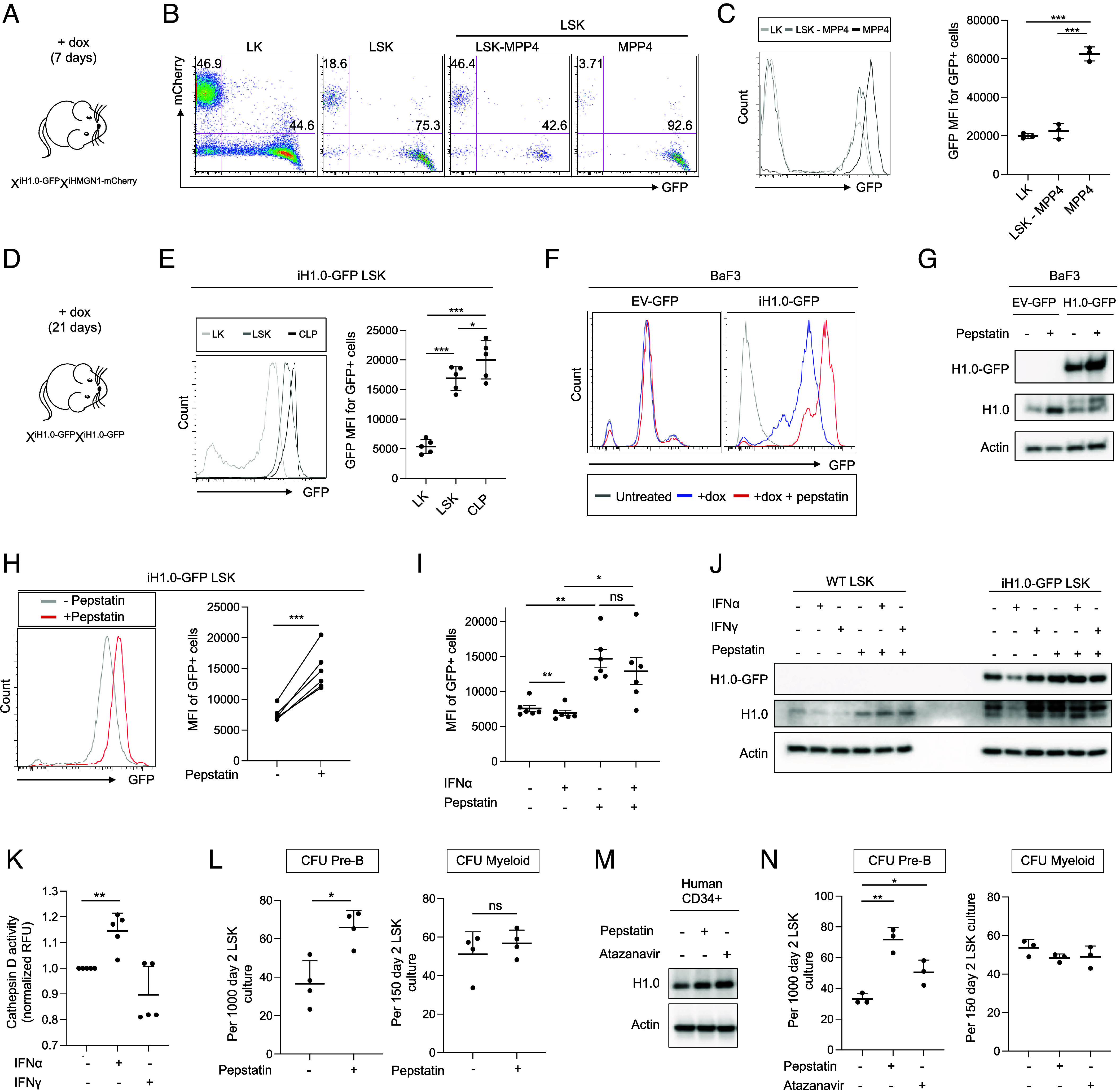
H1.0 level is amenable to physiologic and pharmacologic regulation. (*A*). Scheme of the experimental setup for analyzing bone marrow HSPCs from X^iH1.0-GFP^X^iHMGN1-mCherry^ female mice, which are unmanipulated except for 1 wk of dox water treatment. (*B*). Representative FACS plots showing the distribution of LSK subsets positive for H1.0-GFP or HMGN1- mCherry in mice diagramed in *A*. n = 3 each. (*C*). *Left*: representative FACS histogram showing GFP fluorescence intensity in indicated LSK subsets shown in *B*. *Right*: quantification for GFP mean fluorescence intensity (MFI) for the GFP+ cells in indicated populations. n = 3 each. (*D*). Scheme of the experimental setup for analyzing bone marrow HSPCs from X^iH1.0-GFP^X^iH1.0 GFP^ female mice, which are unmanipulated except for 3 wk of dox water treatment. (*E*). *Left*: representative FACS histogram plot showing the GFP fluorescence intensity in indicated HSPC populations. *Right*: quantification for GFP mean fluorescence intensity (MFI) for the GFP+ cells in indicated populations. n = 5 each. (*F*). Representative FACS histogram showing GFP fluorescence intensity in BaF3 cells expressing viral constructs: control pan-cellular GFP (EV-GFP) and dox inducible H1.0-GFP. Cells were cultured in the presence or absence of pepstatin. See also *SI Appendix*, Fig. S4*C*. (*G*). Representative Western blot using H1.0 and GFP antibodies in BaF3 cells expressing viral EV-GFP or iH1.0-GFP, in the presence or absence of pepstatin. Actin was probed as a loading control. (*H*). Representative FACS histogram plot showing GFP fluorescence intensity in freshly sorted iH1.0-GFP LSK cells cultured in the presence or absence of pepstatin. Right: quantification of GFP mean fluorescence intensity (MFI) in the GFP+ iH1.0 LSK cells. n = 6 each. (*I*). Quantification of GFP MFI in GFP+ iH1.0 LSK cells cultured in the presence or absence of IFNα and pepstatin. n = 6 each. (*J*). Western blot of endogenous H1.0 and H1.0-GFP in WT and iH1.0 + LSK cells cultured in the presence or absence of IFNα/IFNγ ± pepstatin. Actin was probed as a loading control. Results are representative of three independent experiments. (*K*). Cathepsin D activity as measured by a fluorescent substrate. Results are normalized to relative fluorescence units (RFU) in WT LSK cell lysates. n = 5 each. (*L*). Quantification of lymphoid and myeloid CFUs in LSK cells treated with pepstatin. Each dot is an independent experiment. n = 4 each. (*M*). Western blot of endogenous H1.0 protein in human CD34+ cells cultured in the presence of pepstatin or atazanavir. Actin was probes as a loading control. (*N*). Quantification of lymphoid and myeloid CFUs is LSK cells treated with indicated protease inhibitors. Each dot is a triplicate sample; results shown are representative of three independent experiments. n = 3. Individual values as mean ± SD are shown. *P < 0.05, **P < 0.01, ***P < 0.001, by paired, 2- tailed Student’s *t* test, except for 4 N which is unpaired. See also *SI Appendix*, Fig. S4.

The results shown above suggest that H1.0 level might be a regulatory point to adjust the myeloid versus lymphoid lineage output in physiology. We reasoned that changes in H1.0 levels could be reflected by the fluorescence intensity of the H1.0-GFP fusion protein, as shown in Fig. 1 *E*–*G*. In female X^iH1.0-GFP^X^iHMGN1-mCherry^ mice briefly treated with dox water (1 wk) (Fig. 4*A*), most HSPC compartments had roughly equal percentage of cells positive for either transgene, as expected from random X chromosome inactivation (Fig. 4*B* and *SI Appendix*, Fig. S4*A*). However, the lymphoid-biased MPP4 was dominated by H1.0-GFP+ cells, yielding the apparent dominance by H1.0-GFP+ cells within the LSK compartment. Strikingly, among LSK cells, the lymphoid-biased MPP4 display brighter H1.0-GFP (Fig. 4 *B* and *C*). In contrast, HMGN1-mCherry intensity in MPP4 was similar to other LSK subsets (Fig. 4*B* and *SI Appendix*, Fig. S4*B*). On longer treatment with dox water (3 wk), during which H1.0-GFP+ HSPCs presumably underwent further differentiation, the CLPs displayed even brighter H1.0-GFP than the LSK (Fig. 4 *D* and *E*). These results echo the expansion of MPP4-CLP compartments following H1.0 overexpression (Fig. 1 *I* and *M*). Thus, the lymphoid-biased and lymphoid-committed cells display brighter H1.0-GFP, but not HMGN1-mCherry. We interpret these results to indicate potential regulation of H1.0 levels in HSPCs.

Given that proteases regulate HSPC biology (54, 55), we hypothesized that some proteases may be involved in regulating H1.0 levels. We screened a panel of protease inhibitors for those that can increase H1.0-GFP intensity in BaF3 cells expressing dox-inducible H1.0-GFP (iH1.0-GFP) from a retroviral construct. The iH1.0-GFP BaF3 cells were treated with dox plus protease inhibitors, followed by flow cytometry assessment of H1.0-GFP fluorescence intensity (Fig. 4*F* and *SI Appendix*, Fig. S4*C*). Inhibitors of cysteine proteases (E64), cysteine/serine/threonine protease inhibitor (leupeptin), and calpains (calpeptin and PD150606) did not change H1.0-GFP intensity; an inhibitor of serine proteases (pefabloc) had a mild effect, while the aspartyl protease inhibitor pepstatin consistently and significantly increased H1.0-GFP intensity (Fig. 4*F* and *SI Appendix*, Fig. S4*C*). Control BaF3 cells expressing the empty vector (EV) encoding GFP (EV-GFP) had similar fluorescence intensity across all treatment conditions. These results indicate that H1.0-GFP was reduced by aspartyl protease activities. Importantly, pepstatin also increased the endogenous H1.0 protein in BaF3 cells (Fig. 4*G*). We next examined whether pepstatin similarly regulates H1.0-GFP level in LSK cells and found this to be the case (Fig. 4*H*). However, pefabloc had negligible effects on H1.0-GFP intensity in LSK cells, although it had a mild effect in BaF3 cells (*SI Appendix*, Fig. S4 *C* and D), and therefore was not pursued further. We reasoned that the changes in H1.0-GFP fluorescence intensity in LSK cells could serve as a reporter for identifying endogenous regulators of H1.0 in HSPCs.

To this end, we wondered whether inflammatory signals could play a role, as inflammation often leads to myeloid-biased hematopoiesis. We first treated H1.0-GFP+ LSK cells with a panel of immunostimulatory agents and measured their H1.0-GFP intensity (*SI Appendix*, Fig. S4*E*). The results showed that the immunostimulatory agents yielded different H1.0-GFP levels, suggesting signal-dependent H1.0 regulation. Importantly, interferon alpha (IFNα) reproducibly reduced H1.0-GFP intensity and cotreatment with pepstatin blocked this reduction (Fig. 4*I*). Next, we examined whether endogenous H1.0 levels are similarly reduced by IFNα. As shown in Fig. 4*J*, IFNα similarly reduced the endogenous H1.0 level, and this decrease was also blocked by pepstatin in WT LSK cells, paralleling the changes in transgenic H1.0-GFP in LSK cells. This effect was specific for IFNα as IFNγ did not change H1.0 levels. To examine whether IFNα treatment induced aspartyl protease activity, we assessed the proteolytic activity of one of the aspartyl proteases, cathepsin D, in LSK cell lysates, and detected a moderate but consistent increase (Fig. 4*K*). Taken together, H1.0 level in HSPCs can be regulated by IFNα triggered events.

Finally, we examined whether the increased H1.0 level by protease inhibitors could be recapitulated by changes in HSPC lineage fate choices. Consistent with its H1.0-preserving effects, pepstatin increased lymphoid CFU numbers without changing the myeloid CFU of WT LSK cells (Fig. 4*L*), indicating that pharmacologic inhibitors of aspartyl proteases could have lineage modulatory effects on HSPCs. Of note, the HIV protease is an aspartyl protease (56), for which multiple safe and effective inhibitors have been in clinical use. To test the translational possibility that aspartyl protease inhibitors could modulate normal HSPC lineage fate choices, we treated human cord blood CD34+ cells with pepstatin or atazanavir, one of the clinically used HIV protease inhibitors. Both pepstatin and atazanavir increased the endogenous H1.0 protein level (Fig. 4*M*). In agreement with the increased H1.0 level, atazanavir also increased the lymphoid CFU without altering the myeloid CFU of WT LSK cells, similar to pepstatin (Fig. 4*N*). Taken together, our results implicate a potential translational opportunity for aspartyl protease inhibitors to be repurposed to counter the myeloid skewed HSPCs observed during aging and boost their lymphoid potential.

## Discussion

Our results depict a linker histone-driven cell fate regulatory mechanism in multipotent HSPCs. We show that multipotent HSPCs can be programmed to favor the lymphoid fate by increasing the level of a single linker histone, H1.0. These results contrast the observations that lacking single H1 genes is often of little phenotypic consequence (15, 16, 22). It remains an open question whether elevating other H1 isoforms would lead to similar changes in HSPC lineage fate choices. Corroborating a regulatory function, the endogenous H1.0 level changes according to HSPC’s differentiation stage (*SI Appendix*, Fig. S1*P*) and in response to physiological signals, such as IFNα (Fig. 4*J*). Linker histones have been reported to bind to interferon-stimulated genes (ISGs) and suppress their expression (24), whereas H1 depletion can trigger interferon response (25). With our data showing that H1.0 level decreases in response to IFNα (Fig. 4*J*), ISGs could be poised to hyperactivate when H1.0 is low, placing H1.0 and IFNα signaling in a positive feedback loop. This is consistent with the “inflamm-aging” concept where heightened/prolonged interferon signaling accompanies aging and myeloid bias (57–60). In such a model, aspartyl protease inhibitors could potentially break the vicious cycle consisting of inflammation and low H1, which appears to be attainable with pepstatin and a clinically used protease inhibitor atazanavir. Although histone proteolysis has been extensively studied in the formation of Neutrophil Extracellular Trap (NET) or NETosis (61, 62), proteolytic cleavage of core histones can occur without cell death as monocytes differentiate into macrophages (63). To demonstrate HSPC cell-autonomous effects by H1.0, most experiments in this study were performed using transplantation models, which likely provide a more inflammatory microenvironment (64, 65); future studies could dissect the contribution by an inflamed bone marrow environment to the observed phenotype. Outside of hematopoietic lineages, cathepsin L has been reported to cleave H3 during mESC differentiation (66) and cathepsin D cleaves H3 in the involuting mammary gland (67). Defining the mode of H1 downregulation by proteases will substantially add to our understanding of how linker histones integrate with nucleosome/chromatin-based cell fate control.

H1.0’s prolymphoid function is at least in part mediated by chromatin repression of the myeloid driver gene *Hlf*, whose cell type–specific expression pattern may explain the cell context-dependent response to H1.0 overexpression. Specifically, while LSK and GMP similarly express transgenic H1.0 (Fig. 2 *A* and *B*), the cell fate modulatory effect appeared to be much more pronounced in LSK subsets (Fig. 1*M* and *SI Appendix*, Fig. S1*J*) in association with H1.0-mediated repression of *Hlf* (Fig. 3). It remains to be determined whether H1.0 can modulate fate choices in cells whose *Hlf* genomic region is already closed, or via other target genes since many my-HSC genes had reduced chromatin accessibility (Fig. 3 and *SI Appendix*, Fig. S3 and Dataset S2). The chromatin features that mediate the H1 response is surprising, as the most differentially closed chromatin region is 3′ distal to *Hlf* gene—how nucleosome organization and chromatin accessibility at such regions regulate *Hlf* expression remain to be determined. Our data so far point to low GC as a potential genomic feature in the differentially closed chromatin regions. In this regard, we note that Satb1, a protein that binds AT-rich genomic regions, promotes lymphopoiesis (68), raising the possibility of functional cooperation between Satb1 and H1 (69). This feature also implies potential crosstalk with TFs that prefer GC-rich regions, such as CTCF (70). How GC content and nucleosome regulation intersect with lineage instructive TFs in hematopoietic lineage decision also awaits future investigation, with one exciting example offered by PU.1 redistribution-driven myeloid differentiation by an AT-rich minor groove binding drug (71).

## Supplementary Material

Appendix 01 (PDF)

Dataset S01 (XLSX)

Dataset S02 (XLSX)

Dataset S03 (XLSX)

## Data Availability

The data discussed in this publication have been deposited in NCBI’s Gene Expression Omnibus and are accessible through GEO Series accession number GSE309710 (https://www.ncbi.nlm.nih.gov/geo/query/acc.cgi?acc=GSE309710) (72). Previously published data were used for this work (GSE162551) (46). All other data are included in the manuscript and/or supporting information.
