# Supplementary material for: Linker histone regulates the myeloid versus lymphoid bifurcation of multipotent hematopoietic stem and progenitors
Source: Proc Natl Acad Sci U S A. 2025 Oct 21;122(43):e2509412122. doi: 10.1073/pnas.2509412122 (PMC12582276; doi:10.1073/pnas.2509412122)
Supplement: Supplementary file 1 — Appendix 01 (PDF) [file pnas.2509412122.sapp.pdf]

Supporting Information for

Linker histone regulates the myeloid versus lymphoid bifurcation of multipotent hematopoietic stem and progenitors

Kutay Karatepe[1,2*], Bruna Mafra de Faria[1,2*], Jian Zhang[1,2], Xinyue Chen[1,2], Hugo Pinto[3], Dmitry Fyodorov[3], Esen Sefik[4], Michael A. Willcockson[3], Richard A. Flavell[4#], Arthur I. Skoultchi[3] and Shangqin Guo[1,2#]

[1]Department of Cell Biology, Yale University, New Haven, CT 06520
[2]Yale Stem Cell Center, Yale University, New Haven, CT 06520
[3] Department of Cell Biology, Albert Einstein College of Medicine, Bronx, New York 10461
[4] Department of Immunobiology, Yale University, New Haven, CT 06520
[*]These authors contributed equally

[#]Correspondence should be addressed to richard.flavell@yale.edu or shangqin.guo@yale.edu

This PDF file includes:

Supplemental Materials & Methods
Figures S1 to S4
Legends for Datasets S1 to S4
SI References

Other supporting materials for this manuscript include the following:

Datasets S1 to S3

Supplemental Materials & Methods

Mice

Mouse work was approved by the Institutional Animal Care and Use Committee (IACUC). All research animals were housed and maintained in facilities of Yale Animal Resource Center (YARC). *HPRT*::iH1.0-GFP and *HPRT*::iHMGN1-mCherry chimeric mice were generated from passage 0 A2lox.cre H1.0-GFP and HMGN1-mCherry targeted mouse ESCs by Yale Genome Editing Center via blastocyst injection and implantation into C57BL/6J females. High-degree chimeric male offspring were selected by coat color. The iH1.0-GFP and iHMGN1-mCherry alleles were subsequently crossed with B6.SJL-Ptprca Pepcb/BoyJ mice carrying the CD45.1 and *Rosa26*::rtTA allele[1], and backcrossed onto a BL/6 background for at least 8 generations. For all iH1.0-GFP and iHMGN1-mCherry cells, H1.0 and HMGN1-mCherry expression were induced *in vivo* for at least one week by feeding Dox drinking water. For inducible promoter activation, mice were fed drinking water containing 1g/L Doxycycline (Millipore Sigma, D9891) supplemented with 10g/L sucrose (Millipore Sigma, S0389). All experiments, unless otherwise noted, were performed on 8- to 12-week-old age and sex-matched mice.

Bone Marrow Transplantation

Recipient C57BL/6 mice carrying the CD45.2 allele were purchased from Jackson Laboratories and acclimated for 2 weeks. Recipient mice were g-irradiated with one dose of 9.5 Gy. Recipients were injected with 1 x 10^6 BM cells from each donor. Retroorbital bleeding was performed every 4 weeks. BM chimerism was analyzed at the end of each experiment.

Complete Blood Count

Orbital peripheral blood (100 µL) was collected by heparinized capillary tubes (Fisher Scientific, 22-362-566) and transferred into K2-EDTA-coated tubes (Becton Dickinson, 365974). Blood parameters were analyzed by Hemavet 950FS (Drew Scientific).

Cloning and reporter cell lines

H1.0-GFP was a gift from Tom Misteli[2] and HMGN1-GFP was a gift from Michael Bustin[3]. H2B-GFP was a gift from Geoff Wahl[4] (Addgene plasmid # 11680 ; http://n2t.net/addgene:11680 ; RRID:Addgene_11680) and Hlf (TFORF0099) was a gift from Feng Zhang[5] (Addgene plasmid # 141513 ; http://n2t.net/addgene:141513 ; RRID:Addgene_141513). Inducible lentiviral plasmid (pFU-TetO-Gateway-PGK-Puro) was constructed previously[6] by inserting a Gateway cassette (Invitrogen), a PGK promoter and a puromycin resistance gene into the pFU-tetO-Klf4 vector[7] through blunted EcoRI sites. H1.0-GFP, HMGN1-GFP and H2B-GFP were cloned into this destination vector through Gateway recombination. For constitutive expression, sequences were inserted into a pMSCV retroviral backbone[6] again through Gateway recombination. MSCV-IRES-GFP ("empty vector") retroviral expression plasmid was previously described[8].

Viral vectors were transfected in 293T cells using Fugene 6 transfection reagent (Promega, E2691). Viruses harvested from the supernatant were used to transduce BaF3 and LSK cells.

HPRT::iH1.0-GFP and *HPRT*::iHMGN1-mCherry knock-in mouse embryonic stem cells (mESCs) were generated using inducible cassette exchange (ICE) to target the TetO-CMV-H1.0-

GFP or TetO-CMV-HMGN1-mCherry transgene to the *HPRT* locus by cre-recombination in the A2lox.cre mESC cell line[9]. Briefly, H1.0-GFP and HMGN1-mCherry were cloned into P2lox targeting plasmids using HindIII and NotI restriction sites. The targeting plasmids were electroporated into Dox-activated A2lox.cre mESCs. After one day of recovery on neomycin-resistant feeder MEFs, successfully recombined clones were selected by adding 300 ug/ml geneticin (Thermo Fisher, 10131-027) to mESC culture medium. After 6 days of selection, healthy surviving colonies were hand-picked under an inverted microscope and replated onto C57BL/6J irradiated feeder MEFs. These "passage 0" cultures were subsequently split for cryopreservation as well as continued culture and characterization of reporter activity by fluorescence microscopy and flow cytometry.

Flow Cytometry and Cell Sorting

For blood chimerism, red blood cells were lysed by BD FACS Lysing Solution (Becton Dickinson, 349202). Blood cells were incubated with an antibody mixture including antibodies for CD45.1-BV711 (BD, 747742), CD45.2-Pacific Blue (Biolegend, 109820), CD11b-APC/Cy7 (Biolegend, 101226), B220-APC (Biolegend, 103212) and CD3e-BV605 (Biolegend, 100351) on ice for 15 minutes, then washed and filtered before analysis. In some experiments, CD11b-PE (Biolegend, 101207), CD3e-PerCp/Cy5.5 (Biolegend, 100328) or CD3e-Pacific Blue (Biolegend, 100333) were used.

BM cells from femurs and tibias were flushed into Dulbecco's PBS (Life Technologies, 14190-250) supplemented with 2% fetal bovine serum (FBS) (Thermo Fisher, 16000-044). Red blood cells were lysed by ACK buffer (Lonza, BP10-548E). Five million BM cells were first incubated with an antibody mixture containing biotinylated antibodies for mature lineage markers: CD3e-biotin (BD, 553060), CD4-biotin (BD, 553728), CD8a-biotin (BD, 553029), CD11b-biotin (BD, 553309), B220-biotin (BD, 553086), Gr1-biotin (BD, 553125) and Ter119-biotin (BD, 553672) for 15 minutes on ice. After washing, samples were incubated with an antibody mixture including Streptavidin-PerCp (BD, 554064), Sca1-BV605 (Biolegend, 108134), c-Kit-APC (BD, 553356), CD48-BV711 (Biolegend, 103439), CD150-APC/Fire 750 (Biolegend, 115940) and CD135-BV421 (Biolegend, 135314) for LSK subset analysis. Alternatively, CD48-Alexa Fluor 700 (Biolegend, 103425), CD150-PE/Cy7 (Biolegend, 115914) and CD135-PE (Biolegend, 135305) were used in some experiments. For LK subset and CLP analysis, Streptavidin-PerCp (BD, 554064), Sca1-BV605 (Biolegend, 108134), c-Kit-APC (BD, 553356), CD34-Alexa Fluor 700 (BD, 560518), CD16/32-PE/Cy7 (Biolegend, 101318) and CD127-eFluor 450 (Thermo Fisher, 48-1271-82) were used. Samples were incubated on ice for 15 min, then washed and filtered before analysis.

For sorting highly purified cells, an immunomagnetic negative selection kit was used to enrich HSPCs. BM cells were first incubated with an antibody mixture containing biotinylated antibodies for mature lineage markers: CD3e-biotin (BD, 553060), CD4-biotin (BD, 553728), CD8a-biotin (BD, 553029), CD11b-biotin (BD, 553309), B220-biotin (BD, 553086), Gr1-biotin (BD, 553125) and Ter119-biotin (BD, 553672) for 15 minutes on ice. After washing, cells were incubated with Streptavidin microbeads (Miltenyi Biotec, 130-048-101) and passed through LD columns (Miltenyi Biotec, 130-042-901). Enriched cells were stained with Streptavidin-PerCp (BD, 554064), Sca1-BV605 (Biolegend, 108134) and c-Kit-APC (BD, 553356). In some experiments, Sca1-PE (Thermo Fisher, 12-5981-82), CD34-Alexa Fluor 700 (BD, 560518) and

CD16/32-PE/Cy7 (Biolegend, 101318) were used. Cells were sorted on an Aria (Becton Dickinson) cell sorter.

Colony-Forming Unit (CFU) Assays
2 x 10^4 BM cells were seeded in semisolid Methocult GF M3434 medium containing rmSCF, rmIL-3, recombinant human (rh)IL-6, and rhEpo for detection of colony-forming units-granulocyte, monocyte and burst-forming units-erythroid (Stemcell Technologies, 03434). Colony numbers were counted on day 7.

For pre-B colonies, 1 x 10^5 BM cells were seeded in semisolid Methocult M3630 medium containing rhIL-7 (Stemcell Technologies, 03630). Colony numbers were counted on day 10.

Other CFU assays involving mouse LSK cells are described in detail in Cell culture section below.

Proteomics
Mass-spectrometry samples
Crude, whole-cell protein samples were prepared on S-Trap columns (PROTIFI). All reagents, including water, were LCMS grade. Approximately 100,000 WT and iH1.0 LSK cells, as well as reference samples of mouse embryonic stem cells and triple H1 knockout mESCs were lysed in 35 µl Lysis Buffer: 5% SDS, 50 mM triethylammonium bicarbonate (TEAB), pH 8.5. Dithiothreitol (DTT) was added to the final concentration of 20 mM, and the samples were reduced for 10 min at 95°C. Freshly prepared iodoacetamide (IAA, 200 mM in water) was added to 50 mM, and the samples were alkylated for 30 min at room temperature (RT) in the dark. Alkylation was stopped by adding phosphoric acid to 1%. Samples were diluted with S-Trap Buffer (100 mM TEAB) to 165µl, vortexed and loaded onto S-Trap micro spincolumns. Columns were centrifuged for 1 min at 4,000 *g*, and the flow-through was discarded. Columns were then washed four times with 165µl S-Trap Buffer before adding 50 µl Trypsin Digestion Solution (15 µg/ml trypsin in 50 mM TEAB) directly into the columns. Digestions were performed for 1 h at 47°C. Digested peptides were recovered by three successive elutions: first, with 40 µl 50 mM TEAB added directly to the digestion reaction, followed by 40 µl 0.2% formic acid (FA) and finally, with 40 µl 0.2% FA in 50% acetonitrile (ACN). Eluates were pooled, dried completely in a SpeedVac centrifuge (~2 h) and resuspended in 130 µl MS Sample Buffer (0.1% FA, 1% ACN in water).

Instrument settings
LC-MS analyses were performed on a TripleTOF 5600+ mass spectrometer (AB SCIEX) coupled with M5 MicroLC system (AB SCIEX/Eksigent) and PAL3 autosampler. LC separation was performed in a trap-elute configuration, which consists of a trap column (LUNA C18(2), 100 Å, 5 µm, 20 X 0.3 mm cartridge, Phenomenex) and an analytical column (Kinetex 2.6 µm XB-C18, 100 Å, 50 X 0.3 mm microflow coumn, Phenomenex). The mobile phase consisted of water with 0.1% FA (phase A) and 100% ACN containing 0.1% FA (phase B).

Peptides in MS Sample Buffer were injected into a 50-µl sample loop, trapped and cleaned on the trap column with 3% mobile phase B at a flow rate of 25 µl/min for 4 min before being separated on the analytical column with a gradient elution at a flow rate of 5 µl/min. The gradient

was set as follows: 0–24 min: 3% to 35% phase B, 24–27 min: 35% to 80% phase B, 27–32 min: 80% phase B, 32–33 min: 80% to 3% phase B, and 33–38 min at 3% phase B. An equal volume of each sample (30 µl) was injected four times, once for information-dependent acquisition (IDA), immediately followed by DIA/SWATH in triplicate. Acquisitions of distinct samples were separated by a blank injection (80 µl MS Sample Buffer) to prevent sample carryover. The mass spectrometer was operated in positive ion mode with EIS voltage at 5200 V, Source Gas 1 at 30 psi, Source Gas 2 at 20 psi, Curtain Gas at 25 psi, and source temperature at 200°C.

IDA and data analyses
IDA was performed to generate reference spectral libraries for SWATH data quantification. The IDA method was set up with a 200 ms TOF-MS scan from 300 to 1,250 Da, followed by MS/MS scans in a high-sensitivity mode from 100 to 1,500 Da of the top 25 precursor ions above 100 cps threshold (80 ms accumulation time, 100 ppm mass tolerance, rolling collision energy, and dynamic accumulation) for charge states (z) from +2 to +5. IDA files were searched using ProteinPilot (version 5.0.2, ABSciex) with a default setting for tryptic digest and IAA alkylation against a protein sequence database.
The *Homo sapiens* proteome FASTA file (82,493 protein entries, UniProt UP000005640) augmented with sequences for common contaminants was used as a reference for the search. Up to two missed cleavage sites were allowed. Mass tolerance for precursor and fragment ions was set to 100 ppm. A false discovery rate (FDR) of 5% was used as the cutoff for peptide identification.

SWATH acquisitions and data analyses
For SWATH (SWATH-MS, Sequential Window Acquisition of All Theoretical Mass Spectra) acquisitions[10], one 50-ms TOF-MS scan from 300 to 1,250 Da was performed, followed by MS/MS scans in a high-sensitivity mode from 100 to 1,500 Da (15 ms accumulation time, 100 ppm mass tolerance, +2 to +5 z, rolling collision energy) with a variable-width SWATH window[11]. DIA data were quantified using PeakView (version 2.2.0.11391, ABSciex) with SWATH Acquisition MicroApp (version 2.0.1.2133, ABSciex) against selected spectral libraries generated in Protein-Pilot. Retention times for individual SWATH acquisitions were calibrated using 25 or more peptides for plectin (PLEC, UniProt Q15149) and myosin-9 (MYH9, UniProt P35579), two abundant proteins that were highly representative in the IDA ion library and all SWATH acquisitions. The following software settings were utilized: up to 25 peptides per protein, 6 transitions per peptide, 95% peptide confidence threshold, 5% FDR for peptides, XIC extraction window 10 minutes, and XIC width 100 ppm. Protein peak areas were exported as Excel files and processed as described below.

Quantification of proteomics data
Histone H1 peptides were normalized against Histone H4 and nucleosome ratios in WT and iH1.0 LSK cells were modeled using LC-MS data quantifications and known absolute determinations by HPLC of the mouse embryonic stem cell samples[12].

ATAC-seq analysis
LSK cells were sorted from $X^{wt}X^{wt}$ mice and $X^{iH1.0\text{-}GFP}X^{iH1.0\text{-}GFP}$ mice treated with Dox water for 3 weeks. ATAC-seq was performed on 50,000 cells per sample (n = 2 for WT and n = 3 for iH1.0) using a commercially available kit (Active Motif, 53150). Libraries were sequenced on

Illumina HiSeq 2500 platform at Yale Center for Genome Analysis (YCGA). Raw sequencing data have been uploaded to NCBI (GSE309710). Adapter sequences were removed with Trim Galore (http://bioinformatics.babraham.ac.uk./projects/trim_galore). Sequencing reads were aligned to mouse genome (mm10) with Bowtie2[13]. Reads were filtered, sorted and mitochondrial reads were removed with Samtools[14]. Duplicates were removed with Picard (http://broadinstitute.github.io/picard). Sorted .bam files were converted to .bed files with Bedtools[15].

LSK chromatin map was kindly provided by Ross C. Hardison Lab[16]. Briefly, they identified 25 unique chromatin states based on previously published literature. We intersected the ATAC-seq files with the chromatin state file to assign each sequencing read into its unique state using Bedtools[15]. For the calculation of nuclear repeat length (NRL), we used NRLfinder[17], a previously published package that contains custom Phyton scripts. For additional information, please see the full package, available at https://github.com/tommyjohn21/nrl_finder. Based on their NRL properties, we combined 25 different states into 3 types. Type A is open chromatin (has no NRL) in both WT and iH1.0 LSK cells. Type B exhibits NRL in both but NRL increases in iH1.0 LSK cells. Importantly, type C has no NRL in WT LSK cells and exhibits an NRL of 190 bp in iH1.0 LSK cells. Then, GC content of each region in 25 chromatin states was determined using mm10 genome and nuc -fi function in Bedtools[15]. Violin plots and median value for each state's GC content were determined by custom R scripts. Codes available upon request.

To calculate differential peak accessibility and call peaks, MACS2 BAMPE mode was used[18]. Differentially opened and closed regions (DOCRs) were analyzed using Diffbind default settings and visualizations were done using deepTools[19] (version 2.5.7). Motif of TFBSs enrichment of closed to open (CO) and open to closed (OC) in iH1.0 LSK compared to WT LSK cells were performed using HOMER (http://homer.ucsd.edu/homer/). GO analysis of DOCRs was performed using GREAT (http://great.stanford.edu/public/html/index.php). Coverage tracks (bigWig format) were generated with bamCoverage from deepTools. Publicly available GSE162551 dataset was used demonstrate Hlf accessibility in different HSPC subsets along with WT and iH1.0 LSK cells[20]. Similarly, publicly available H3K4me3 ChIP-seq data from GSE59636 dataset was used to assess an active epigenetic mark in different HPSC subsets[21].

qPCR

To assess *Hlf* accessibility in iH1.0 LSK cells, we performed qPCR on DNA samples submitted to ATAC-seq using iQ SYBR Green Supermix (Biorad, 170-8882), mRPL30 primers (Cell Signaling Technology, 7015S) and the below primers:

Forward primer for the 3' UTR region: 5'-GCTGCAGAACATTTAGCCCA-3'
Reverse primer for the 3' UTR region: 5'-TGGAATGGATGCTTGGGAGT-3'
Forward primer for the enhancer region in 2nd intron: 5'-GACCAGAGCTCACAACCACA-3'
Reverse primer for the enhancer region in 2nd intron: 5'-AGGGTGCTTTGTTGTCCTCT-3'

To assess *Hlf* mRNA expression levels, RNA was isolated from fluorescence-activated

cell sorted (FACS) LSK cells from WT mice and iH1.0 mice treated with Dox by the phenol-chloroform method using Trizol. cDNA was synthesized using SuperSript III First-strand Synthesis Supermix (Thermo Fisher Scientific, 18080-400). Quantitative RT-PCR was performed using iQ SYBR Green Supermix (Bio-rad, 170-8882) and Bio-Rad CFX384 Real-Time PCR System.. Primers used are listed below: *Hlf*_fwd: 5'-GACCCACCTTATGGGACAAA-3'; *Hlf*_rev: 5'-GGATGCCATTCTCTGACAGG-3'; *H1.0*_fwd: 5'-TTGTCCATCAAGCGCCTAGT-3'; *H1.0*_rev: 5'-TGGCCACTTTCTTGACTTCC-3'; *Gapdh*_fwd: 5-GGTGCTGAGTATGTCGTGGAG-3'; *Gapdh*_rev: 5'-GGCGGAGATGATGACCCTTT-3'. Primers for *Hlf* were taken from a previously published paper[22].

Cell culture

293T cells were cultured in a standard growth medium consisting of DMEM basal medium (Thermo Fisher, 11995-065) with 10% heat-inactivated FBS (Thermo Fisher, 16000-044) and 1% penicillin/streptomycin/L-glutamine supplement (Thermo Fisher, 10378-016).

BaF3 cells were cultured in an RPMI based growth medium (Thermo Fisher, 11875-093) supplemented with 10% heat-inactivated FBS, 1% penicillin/streptomycin/L-glutamine and 270 pg/ml mIL-3 (Peprotech, 213-13). For protease inhibitor studies with BaF3 cells, cells were treated with 20 μM pepstatin (Millipore Sigma, PEPS-RO 10253286001), 30 μM E64 (Millipore Sigma, 11206893001), 100 μM leupeptin (Millipore Sigma, 11206893001), 50 μM calpeptin (Millipore Sigma, 03-34-0051) and 25 μM PD150606 (Millipore Sigma, D5946) for 2 days in the presence of Dox.

Primary LSK cells were cultured in X-Vivo 15 medium (Lonza, 04-418Q) supplemented with 1% BSA (Stemcell Technologies, 09300), 1% penicillin/streptomycin/L-glutamine, 0.14 μl/ml B-mercaptoethanol (Sigma, M3148), 100 ng/ml mSCF (Peprotech, 250-03), 50 ng/ml mTPO (Peprotech, 315-14), 50 ng/ml Flt3L (Peprotech, 250-31L) and 20 ng/ml IL-3. For inducible promoter activation *in vitro*, doxycycline (Millipore Sigma, D9891) was added to cell culture medium at 2 μg/ml.

For most experiments involving protease inhibitors and immunostimulatory agents, mouse LSK cells were first treated with dox for 2 days. Then, cells were cultured in the presence or absence of 20 μM pepstatin (Millipore Sigma, PEPS-RO 10253286001) for another 2 days. Similarly, for immunostimulatory agents, cells were treated with 100 ng/ml IFNα (Biolegend, 752802), 20 ng/ml IFNγ (Peprotech, 315-05), 10 μg/ml LPS (Millipore Sigma, L4516), 1 μg/ml Pam3CSK4 (R&D Systems, 4633), 25 ng/ml IL1b (Peprotech, 211-11B) or 50 ng/ml TNFa (Peprotech, 315-01A) for another 2 days in the presence of dox.

For pepstatin and atazanavir-regulated CFU assays, LSK cells were cultured in the presence or absence of 20 μM pepstatin or 8μM atazanavir in X-Vivo 15-based medium described above. On day 2, cells were counted. 1,000 cells/well were seeded in M3630 medium for pre-B colonies and 150 cells/well were seeded in M3434 medium for myeloid colonies in the presence or absence of 20 μM pepstatin or 8μM atazanavir. Pre-B colonies were counted after 5 days in M3630 medium and myeloid colonies were counted after 7 days in M3434 medium.

For cathepsin D activity, WT LSK cells were cultured for 2 days, split and cultured in the presence or absence of IFNα or IFNγ for 1 more day. Then, cells were counted and equal number of cells was used to test cathepsin D activity with a commercially available fluorometric assay kit (Abcam, ab65302) according to manufacturer's recommendations.

Mobilized CD34+ adult blood cells were purchased from Yale Cooperative Center of Excellence in Hematology. Cells were cultured in Stemspan SFEM II (Stemcell Technologies, 09655) supplemented with 100 ng/ml hSCF (Peprotech, 300-07), 100 ng/ml hTPO (Peprotech, 300-18), 100 ng/ml hFlt3L (300-19), 5 ng/ml hIL3 (Peprotech, 200-03), 20 ng/ml hIL6 (Peprotech, 200-06) and 0.75 μM SR1 (Stemcell Technologies, 72342). Cells were cultured in the presence or absence of 20 μM pepstatin, 20 μM atazanavir (Cayman Chemicals, 11733) or 1 U/μl hIFNa2b (Invivogen, rcyc-hifna2b) for 24 hours.

HSPC transduction for CFU assays
Freshly sorted LSK cells from WT mice were transduced with EV-GFP, H1.0-GFP and Hlf in the presence of polybrene (Millipore Sigma, TR1003G) on retronectin (Takara Bio, T100B) coated plates. For the cells that were transduced with both H1.0-GFP and Hlf, cells were first transduced Hlf. Next day, cells were transduced with H1.0-GFP. On day 2, H1.0-GFP transduction efficiency was confirmed by microscopy and 2 μg/ml puromycin (Thermo Fisher, A11138-03) was added to cells transduced with Hlf for drug selection. On day 4, cells were counted. 2,000 cells/well were seeded in M3630 medium for pre-B colonies and 150 cells/well were seeded in M3434 medium for myeloid colonies. Colonies were counted after 7 days in M3630 and M3434 medium.

Western blotting
The following primary antibodies were used: H1.0 (Santa Cruz, sc-56695), GFP (Abcam, ab290) and pan-actin (Abcam, ab6276).

Fig3 schematic created in BioRender. Guo, S. (2025) https://BioRender.com/7m91pn1

Figure S1. H1.0 expression in HSPCs confers increased lymphopoiesis.

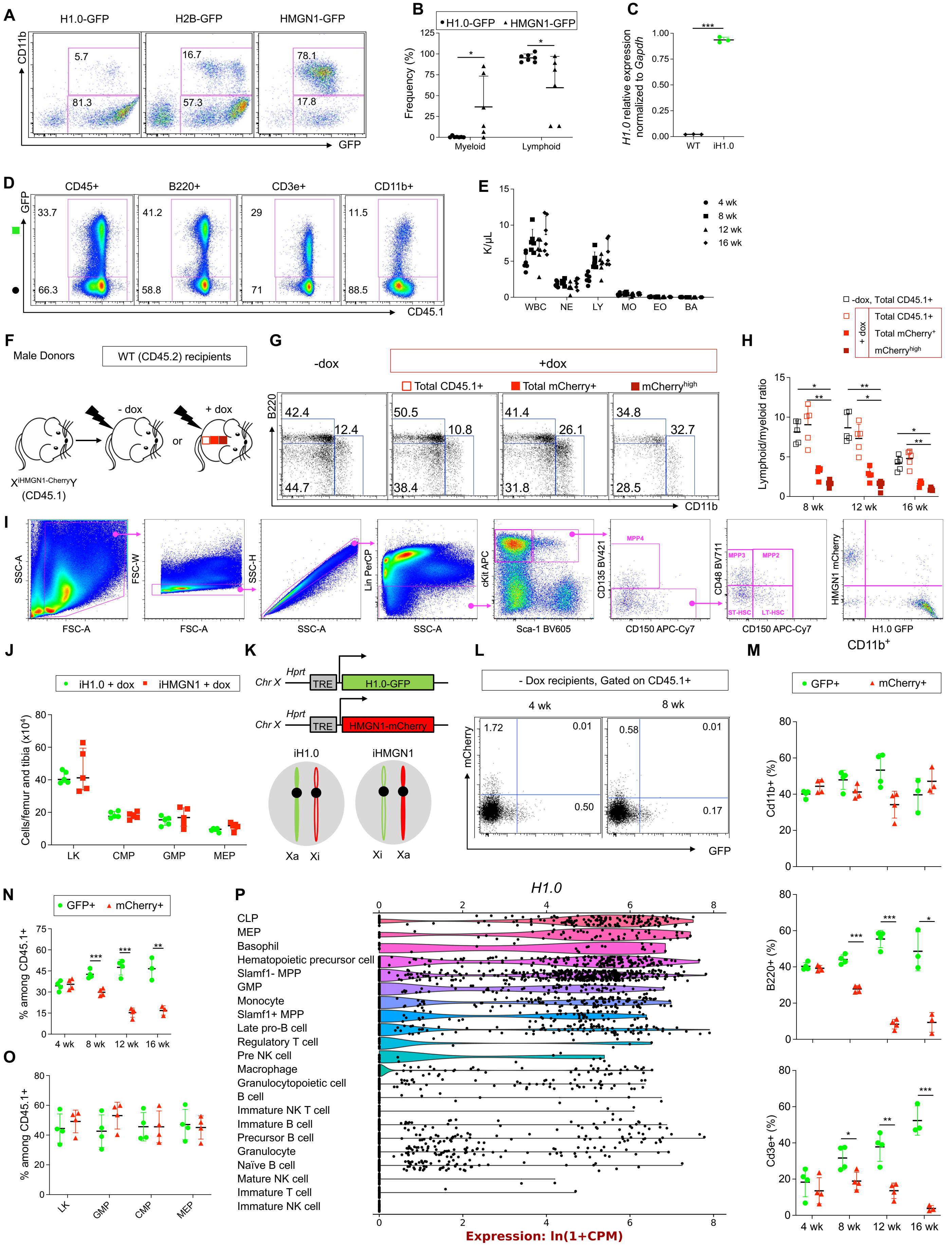

Figure S1. H1.0 expression in HSPCs confers increased lymphopoiesis *in vivo*.
(A) Representative FACS dot plots for donor-derived peripheral blood cells at 8 weeks post- transplantation with LSK cells transduced with lentiviral H1.0-GFP, H2B-GFP and HMGN1-GFP.
(B) Quantification of lineage marker+ cells within donor-derived GFP+ cells in recipients reconstituted with H1.0-GFP or HMGN1-GFP-transduced LSK cells at 16 weeks post- transplantation. n=6-7 per group.
(C) *H1.0* mRNA level in freshly sorted LSK cells from WT ($X^{wt}X^{wt}$) and $X^{iH1.0\text{-}GFP}X^{iH1.0\text{-}GFP}$ mice treated with dox water for 3 weeks. n = 3 per group.
(D) Related to Fig 1B-D. Representative FACS dot plots of peripheral blood B220+ B cells, CD3+ T cells and CD11b+ myeloid cells showing the GFP- and GFP+ cell percentage in the indicated lineage marker+ populations at 16 weeks post-transplantation.
(E) Related to Fig 1B-D. Complete blood cell (CBC) counts including white blood cell, neutrophil, lymphocyte, monocyte, eosinophil and basophil counts in WT recipients reconstituted with $X^{wt}X^{iH1.0\text{-}GFP}$ WBM 4-16 weeks post-transplantation. All recipients were maintained on dox water. n = 6 per group.
(F) Scheme of the experimental setup for noncompetitive WBM transplantation using $X^{iHMGN1\text{-}mCherry}Y$ male donors. The recipients are treated with regular or dox water. In the dox water treated recipients, donor (CD45.1+) cells are further divided by their HMGN1-mCherry fluorescence intensity.
(G) Representative FACS dot plots for the recipients shown in S1F, at 16 weeks post- transplantation. – dox and + dox total CD45.1+, total Cherry+ and $Cherry^{high}$ cells in peripheral blood stained for lineage markers are shown. $Cherry^{high}$ denotes the top 10% among total mCherry+ cells.
(H) Quantification of the lymphoid/myeloid ratios in the recipients shown in S1G.
(I) Representative FACS dot plots and gating strategy for HSPC subsets.
(J) Related to Fig 1H-I. Quantification of the number of myeloid committed progenitors per leg (femur and tibia) in recipients at 16 weeks post-transplantation. n = 5 per group.
(K) Related to Fig 1J. Diagram for comparing the H1.0-GFP+ and HMGN1-mCherry+ cells within the same recipients, engrafted with $X^{iH1.0\text{-}GFP}X^{iHMGN1\text{-}mCherry}$ donor cells. Lower diagram illustrates active X (Xa) and inactive X (Xi) chromosomes. When recipients are treated with dox water, all donor derived cells express either H1.0-GFP or HMGN1-mCherry, but not both, due to random X- chromosome inactivation.
(L) Related to Fig 1K. Representative FACS dot plots showing the donor derived (CD45.1+) peripheral blood cells at 4 and 8 weeks post-transplantation in recipients on regular water.
(M) Related to Fig 1L. Quantification for %H1.0-GFP+ and %mCherry+ cells among CD11b+ myeloid, B220+ B, CD3e+ T cells in the peripheral blood of recipient mice at 4, 8, 12 and 16 weeks post-transplantation. n = 4 for 4-12 weeks and n = 3 for 16 weeks.
(N) Related to Fig 1L. Quantification for %H1.0-GFP+ and %mCherry+ cells among donor derived (CD45.1+) cells in peripheral blood of recipient mice at 4, 8, 12 and 16 weeks post-transplantation. n = 4 for 4-12 weeks and n = 3 for 16 weeks.
(O) Related to Fig 1M. Quantification of bone marrow myeloid progenitor subsets positive for H1.0-GFP or HMGN1-mCherry in recipient mice at 12-16 weeks post- transplantation. n = 4 each.
(P) Violin plot of single cell RNA-seq results depicting *H1.0* mRNA levels in bone marrow hematopoietic cells according to the Tabula Muris database.

Individual values as well as means ± SD are shown. *p<0.05, **p<0.01, ***p<0.001, by unpaired, 2-tailed Student’s t-test except for H (values as median ± interquartile range and statistical analysis Kruskal-Wallis). See also Figure 1.

Figure S2. H1.0 overexpressing HSPCs display strengthened nucleosome organization

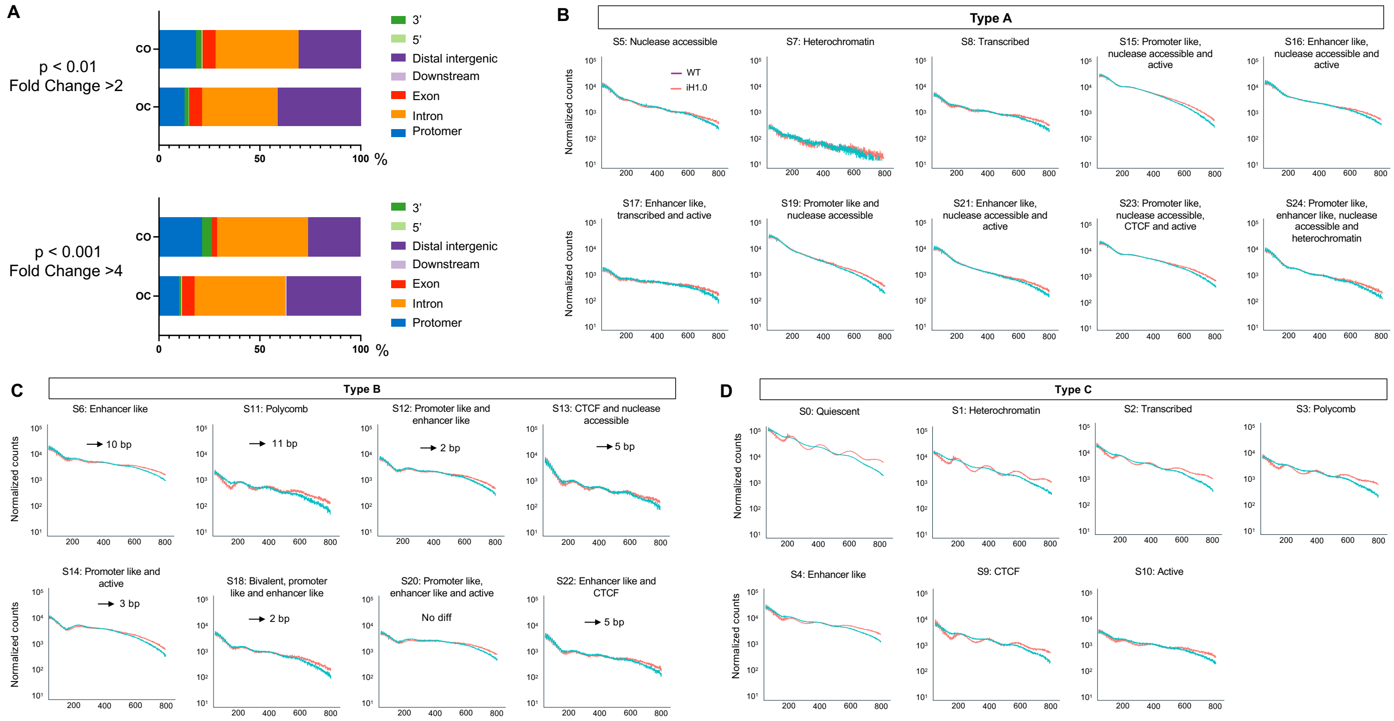

Figure S2. H1.0-overexpressing HSPCs display strengthened nucleosome organization.

(A) Differentially accessible chromatin regions between WT and iH1.0+ LSK cells contain similar genomic annotations. CO: regions more open in iH1.0+ LSK cells; OC: regions more closed in iH1.0+ LSK cells. Two levels of cutoff stringency yield similar results. (B-D) ATAC-seq fragment lengths and density in WT and iH1.0+ LSK cells across all 25 ChromHMM annotated chromatin state, as described by Xiang et. al. 2024. Changes in nucleosome repeat length (right shift) was calculated using the "NRLfinder" as described by Willcockson et. al. 2021 and indicated on each plot with an arrow indicating the magnitude of change. Plots are grouped according to their response type upon iH1.0 induction: unaffected (type A), right shift (type B), gaining nucleosome repeat signal with iH1.0 (type C). See also Figure 2 and Table S3.

Figure S3. **H1.0 expression imparts lymphoid fate potential by reducing chromatin accessibility and gene expression of *Hlf*.**

A

Seqnames	BaseMean	log2FC	padj	Annotation	Gene Symbol	Change
chrX	347.53	-12.25	3.41E-21	Promoter	Hprt	OC
chr6	96.19	-2.99	8.13E-18	Intron	Tspan9	OC
chr11	114.43	-2.84	1.49E-17	Distal Intergenic	**Hlf**	OC
chr8	183.41	-2.12	1.78E-17	Distal Intergenic	Gm21119	OC
chr2	91.72	-2.77	1.06E-16	Promoter	Tubb1	OC

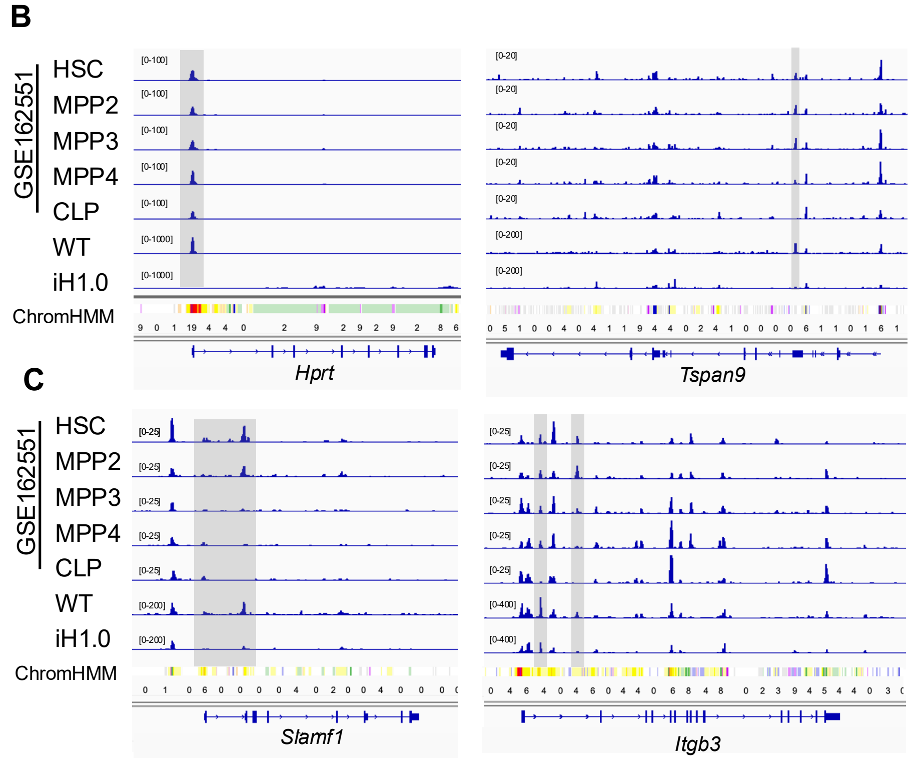

D

	Start (Chr: nucleotide)	End (Chr: nucleotide)	GC%
Hlf – gene body	11: 90336535	11: 90390916	46.73%
Hlf – H1.0 site in intergenic region	11: 90333838	11: 90335192	46.57%

Figure S3. H1.0 expression imparts lymphoid fate potential by reducing chromatin accessibility and gene expression of *Hlf*.

(A) Top 5 most differentially closed chromatin regions in iH1.0+ as compared to WT LSK cells, ranked by statistical significance and effect size.

(B) Related to Fig 3A. Chromatin accessibility measured by ATAC-seq around the genomic regions of *Hprt* and *Tspan9* during WT HSPC differentiation from dataset GSE162551, aligned with those obtained from WT and iH1.0 LSK cells, further aligned with ChromHMM chromatin states. n = 2 for WT and n = 3 for iH1.0+ LSK cells; one representative shown for each genotype.

(C) Related to Fig 3A. Chromatin accessibility measured by ATAC-seq around the genomic regions of *Slamf1* and *Itgb3* during WT HSPC differentiation from dataset GSE162551, aligned with those obtained from WT and iH1.0 LSK cells, further aligned with ChromHMM chromatin states. n = 2 for WT and n = 3 for iH1.0+ LSK cells; one representative shown for each genotype.

(D) Related to Fig 3A, 2D. GC content of *Hlf* gene body and the differentially accessible intergenic region shown in Fig 3A.

Individual values as well as means ± SD are shown. $^{*}p<0.05$, $^{**}p<0.01$, $^{***}p<0.001$, by unpaired, 2-tailed Student's t-test. See also Figure 3.

Figure S4. H1.0 level is amenable to physiologic and pharmacologic regulation.

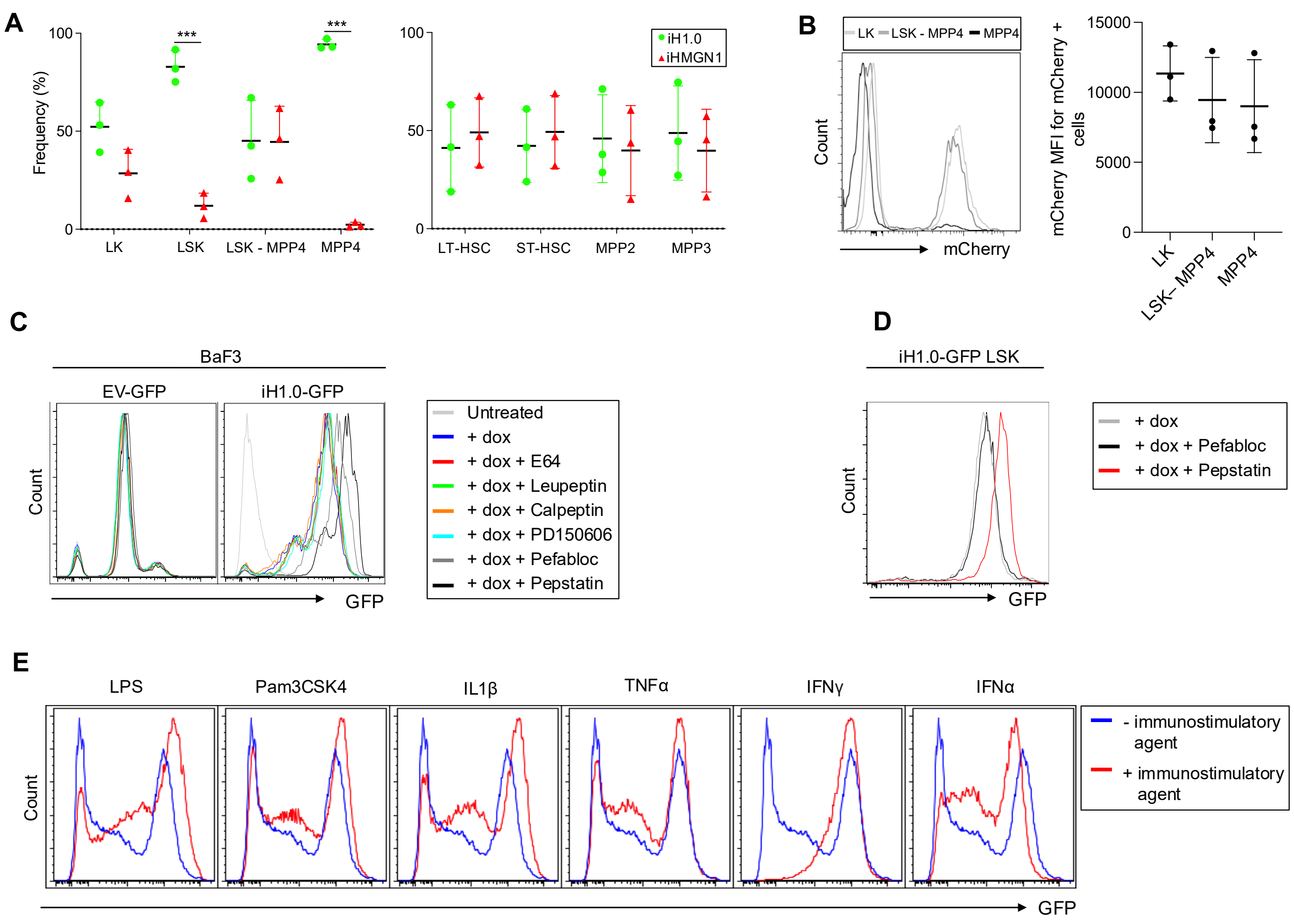

Figure S4. H1.0 level is amenable to physiologic and pharmacologic regulation.

(A) Quantification for the distribution of LSK subsets positive for H1.0-GFP or HMGN1-mCherry from $X^{iH1.0\text{-}GFP}X^{iHMGN1\text{-}mCherry}$ female mice, which are unmanipulated except for one week of dox water treatment. n = 3 each. Related to Fig 4A-C.

(B) Left: representative FACS histogram showing mCherry fluorescence intensity in indicated HSPC populations. Right: quantification for mCherry mean fluorescence intensity (MFI) for mCherry+ cells in indicated populations. n = 3.

(C) Representative FACS histogram showing GFP fluorescence intensity in BaF3 cells expressing viral constructs: control pan-cellular GFP (EV-GFP) and dox inducible H1.0-GFP. Cells were cultured in the presence or absence of protease inhibitors, including E64, leupeptin, calpeptin, PD150606, pefabloc and pepstatin. Results are representative of two independent experiments. See also Fig 4F.

(D) Representative FACS histogram showing GFP fluorescence intensity in freshly sorted iH1.0-GFP LSK cells cultured in the presence or absence of pepstatin and pefabloc. Results are representative of six independent experiments. All cultures have dox added.

(E) Representative FACS histogram showing GFP fluorescence intensity in freshly sorted iH1.0-GFP LSK cells cultured in the presence or absence of indicated immunostimulatory agent. All cultures have dox added. Results are representative of at least two independent experiments.

Individual values as means ± SD are shown. *$p<0.05$, **$p<0.01$, ***$p<0.001$, by paired, 2- tailed Student's t-test. See also Figure 4.

Dataset S1 (separate file): Proteomic based quantification of individual H1 isoform abundance ralative to total H1 in WT and iH1.0-GFP+ LSK and GMP cell extracts. Related to Fig 2.

Dataset S2 (separate file): Differentially open and closed regions from ATAC-seq data between WT and iH1.0-GFP+ LSK.

Individual tabs list the total ATAC-seq peaks and significantly different peaks called with different stringency, either $p < 0.01$ and fold change of >2 or $p < 0.001$ and fold change of >4. CO: more open in H1.0-GFP+ LSK; OC: more closed in H1.0-GFP+ LSK.

Dataset S3 (separate file): Details of the ATAC-seq reads numbers, percentage across 25 chromatin states defined by ChromHMM and classification into three chromatin types.

SI References

1 Hochedlinger, K. *et al.* Ectopic expression of Oct-4 blocks progenitor-cell differentiation and causes dysplasia in epithelial tissues. *Cell* (2005).

2 Misteli, T. *et al.* Dynamic binding of histone H1 to chromatin in living cells. *Nature* (2000).

3 Lim, J. H. *et al.* Chromosomal protein HMGN1 modulates histone H3 phosphorylation. *Mol Cell* (2004).

4 Kanda, T. *et al.* Histone-GFP fusion protein enables sensitive analysis of chromosome dynamics in living mammalian cells. *Curr Biol* (1998).

5 Joung, J. *et al.* A transcription factor atlas of directed differentiation. *Cell* (2023).

6 Guo, S. *et al.* Complex oncogene dependence in microRNA-125a-induced myeloproliferative neoplasms. *Proc Natl Acad Sci U S A* (2012).

7 Stadtfeld, M. *et al.* Defining molecular cornerstones during fibroblast to iPS cell reprogramming in mouse. *Cell Stem Cell* (2008).

8 Lu, J. *et al.* MicroRNA-mediated control of cell fate in megakaryocyte-erythrocyte progenitors. *Dev Cell* (2008).

9 Iacovino, M. *et al.* Inducible cassette exchange: a rapid and efficient system enabling conditional gene expression in embryonic stem and primary cells. *Stem Cells* (2011).

10 Zhu, X. *et al.* Comparison of information-dependent acquisition, SWATH, and MS(All) techniques in metabolite identification study employing ultrahigh-performance liquid chromatography-quadrupole time-of-flight mass spectrometry. *Anal Chem* (2014).

11 Zhang, Y. *et al.* The Use of Variable Q1 Isolation Windows Improves Selectivity in LC-SWATH-MS Acquisition. *J Proteome Res* (2015).

12 Yang, S. M. *et al.* H1 linker histone promotes epigenetic silencing by regulating both DNA methylation and histone H3 methylation. *Proc Natl Acad Sci U S A* (2013).

13 Langmead, B. *et al.* Fast gapped-read alignment with Bowtie 2. *Nat Methods* (2012).

14 Li, H. *et al.* The Sequence Alignment/Map format and SAMtools. *Bioinformatics* (2009).

15 Quinlan, A. R. *et al.* BEDTools: a flexible suite of utilities for comparing genomic features. *Bioinformatics* (2010).

16 Xiang, G. *et al.* Interspecies regulatory landscapes and elements revealed by novel joint systematic integration of human and mouse blood cell epigenomes. *bioRxiv* (2023).

17 Willcockson, M. A. *et al.* H1 histones control the epigenetic landscape by local chromatin compaction. *Nature* (2020).

18 Zhang, Y. *et al.* Model-based analysis of ChIP-Seq (MACS). *Genome Biol* (2008).

19 Ramirez, F. *et al.* deepTools: a flexible platform for exploring deep-sequencing data. *Nucleic Acids Res* (2014).

20 Itokawa, N. *et al.* Epigenetic traits inscribed in chromatin accessibility in aged hematopoietic stem cells. *Nat Commun* (2022).

21 Lara-Astiaso, D. *et al.* Immunogenetics. Chromatin state dynamics during blood formation. *Science* (2014).

22 Wahlestedt, M. *et al.* Critical Modulation of Hematopoietic Lineage Fate by Hepatic Leukemia Factor. *Cell Rep* (2017).
